# Cell type-dependent induction of type I interferon and PARP1 activation in astrocytes and neurons during chikungunya virus infection

**DOI:** 10.1128/spectrum.04178-25

**Published:** 2026-06-24

**Authors:** Lisa Pieterse, Taewoo Kim, Che-Yuan Chang, Ilsa T. Kirby, Benjamin H. Nguyen, Larissa Cortes Morales, Rodney Eric Williams, Easwaran Sreekumar, Michael S. Cohen, Anthony K. L. Leung, Diane E. Griffin, Rachy Abraham

**Affiliations:** 1 W. Harry Feinstone Department of Molecular Microbiology and Immunology, Bloomberg School of Public Health, Johns Hopkins University 1466, Baltimore, Maryland, USA; 2 Department of Biochemistry and Molecular Biology, Bloomberg School of Public Health, Johns Hopkins University 1466, Baltimore, Maryland, USA; 3 Department of Physiology and Pharmacology, Oregon Health & Science University 6684, Portland, Oregon, USA; 4 Department of Molecular Biology and Genetics, Johns Hopkins University 1466, Baltimore, Maryland, USA; 5 McKusick-Nathans Department of Genetic Medicine, Johns Hopkins University 1466, Baltimore, Maryland, USA; 6 Department of Oncology, School of Medicine, Johns Hopkins University 1466, Baltimore, Maryland, USA; National University of Singapore, Singapore, Singapore

**Keywords:** astrocytes, neurons, chikungunya virus, ADP ribosylation, innate immunity, PARPs, PARP1, RIG-I-like receptors

## Abstract

Chikungunya virus, a mosquito-borne alphavirus, causes fever, rash, arthritis, and neurological disorders. Its non-structural protein 3 harbors a macrodomain, a key neurovirulence factor that removes adenosine diphosphate ribose from ADP-ribosylated substrates. Notably, chikungunya virus infection results in distinct ADP-ribosylation patterns and non-structural protein 3 macrodomain-mediated replication dynamics in astrocytes and neurons. Understanding the connection between ADP-ribosylation and the activation of innate immunity, particularly interferon release, is key to elucidating how the cellular immunological state influences ADP-ribosylation, an understudied post-translational modification during viral infection. Here, murine astrocytic (C8-D1A) and neuronal (NSC-34) cells were infected with chikungunya virus to profile transcript and protein expression of innate immune mediators and type I IFNs. The role of PARP1 in global ADP-ribosylation patterns was assessed using PARP-specific inhibitors and genetic depletion approaches. Our investigations revealed that neuronal chikungunya virus infection induces ADP-ribosylation through PARP1 activation, driven by caspase-3-mediated apoptosis, without transcriptionally activating PARPs. In contrast, astrocytic infections showed minimal ADP-ribosylation despite transcriptional activation of interferon-stimulated PARPs. Neurons exhibited limited innate immune response gene transcriptional activity, whereas astrocytes demonstrated strong upregulation of genes essential for pattern recognition receptor activation, thus enhancing double-stranded RNA sensing and increasing type I interferon production during infection. We posit that PARP1 activation and type I IFN response differentially regulate ADP-ribosylation in chikungunya virus-infected neural cells in a cell type-dependent manner.

**IMPORTANCE:**

Chikungunya virus is an emergent mosquito-borne alphavirus increasingly associated with neurological infection and subsequent long-term disabilities. Its continued global spread and recurrent outbreaks underscore its significant pandemic potential and the urgent need for effective countermeasures. Chikungunya virus showcases distinct, cell-type dependent replication dynamics within astrocytes and neurons, two major permissive cerebral cell types. However, understanding of the immunological basis of such cell type-specific infection dynamics remains limited, yet is necessary to elucidate virus pathogenesis within the brain and thus identification of downstream drug targets. Our study characterized two distinctly activated innate immunological pathways in chikungunya virus-infected astrocytes versus neurons, thus significantly contributing to molecular understanding cell type-specific chikungunya virus neurovirulence on a molecular level.

## INTRODUCTION

Chikungunya virus (CHIKV), an alphavirus transmitted by mosquitoes, was first isolated in East Africa. Since its discovery, CHIKV has spread from Africa to Asia, Europe, and the Americas, causing large outbreaks characterized by fever, maculopapular rash, arthritis, and neurological sequelae. CHIKV has diversified into three main genotypes, including the Asian, East/Central/South African, and West African genotypes that are further divided into sub-lineages (1–3). Concerningly, infection with the Asian clade is increasingly associated with severe neurological sequelae such as encephalitis, myelitis, encephalopathy, Guillain–Barré syndrome, and meningoencephalitis (4–12). CHIKV infection of the central nervous system (CNS) is associated with high mortality (6%–11%) and morbidity rates (94%), the latter due to chronic, disabling manifestations, including clinical depression, cognitive decline, fatigue, general immobility, and hand inflexibility (11, 13–16). Recent encephalitis outbreaks indicate that CHIKV significantly contributes to neurological diseases in the Indian subcontinent and the Americas (11, 17–21). Furthermore, enhanced CHIKV adaptation to urban transmission cycles, expanded vector distribution (1, 2), and the continued lack of effective therapeutic interventions or widespread vaccine deployment have collectively increased the risk of altered clinical outcomes, including neurological complications, thus underscoring the urgent need for the development of intervention strategies. Although Vimkunya, a virus-like particle vaccine, has recently been licensed by the U.S. Food and Drug Administration, the vaccine has yet to undergo confirmatory efficacy clinical trials. Furthermore, the rollout Vimkunya has not been a major public health focus, as vaccine production is still limited and costly. Ixchiq, a single-dose, live attenuated vaccine, is no longer approved in the U.S. due to serious adverse events. Treatment options for CHIKV disease remain limited (22).

CHIKV, like other alphaviruses, possesses a highly conserved macrodomain (MD) in the 5′ terminal of non-structural protein 3 (nsP3), which acts as a determinant of neurovirulence in mice and neurons *in vitro* (23–26). MDs are characterized by their ability to bind ADP-ribose (ADPr) and its derivatives, as well as the capacity to remove ADPr from proteins (26–28). Such ADPr binding and hydrolase activities are central to ADP-ribosylation, a post-translational modification (PTM) that a plays crucial role in cellular processes such as DNA repair, condensate formation, chromatin remodeling, NF-κB activation, and cell death pathways (29). ADP-ribosylation is mediated by ADP-ribosyltransferases (ARTs) (30), which include a 17-member subgroup previously known as poly-ADP polymerases, or PARPs, in humans. PARPs transfer ADPr moieties from nicotinamide adenine dinucleotide (NAD+) to target proteins on various amino acids, resulting in either mono-ADP-ribosylation (MARylation) or poly-ADP-ribosylation (PARylation) of substrates, depending on the H-Y-E motif in the catalytic domain (31). Most genes encoding mono-ARTs are regulated by interferon (IFN) (30–35), while poly-ARTs are not responsive to IFN (36, 37).

Our previous studies have shown that the inhibition of ART activity significantly reduces alphavirus replication in neurons (24). Mutations in the CHIKV MD that severely impair ADPr binding and hydrolase activity result in non-viable viruses, underscoring the indispensable role of the MD in RNA synthesis. Viruses with reduced ADPr-binding or hydrolase activity exhibit impaired replication and decreased virulence in mice (23–25). Most importantly, mutants with deficient ADPr-binding and hydrolase capacities have cell type-dependent effects on the translation of subgenomic RNA, and thus production of structural proteins, in neurons and astrocytes (38). Furthermore, unlike most arboviruses associated with encephalitis, CHIKV preferentially infects astrocytes, key immunomodulators of the CNS (39), rather than neurons, in human and murine brains, as well as in culture (38, 40–43). These findings underscore the importance of investigating the immunological responses and ADP-ribosylation events occurring within different CNS cells in order to elucidate the cell type-dependent functions of the alphavirus MD.

Upon entry of a positive-sense, single-stranded RNA (ssRNA) virus into the host, germline-encoded pattern recognition receptors (PRRs) such as Toll-like receptors (TLRs) and/or retinoic acid-inducible gene I (RIG-I/DDX58)-like receptors (RLRs) are activated. Such activation involves transcription factors such as IFN regulatory factor 3 (IRF3) and/or IRF7 (44–47). Once phosphorylated, IRF3 forms homodimers or heterodimers with IRF7, translocates to the nucleus, and induces type I IFN expression (47, 48). Released IFNs bind to cell-surface receptors and activate the Janus kinase/signal transducer and activator of transcription pathway, leading to the transcription of hundreds of IFN-stimulated genes (ISGs), including those encoding *Parp*s (48). While RIG-I and TLR3 have been identified as critical for CHIKV infection, the roles of MDA5/IFIH1 and TLR7 remain unclear, despite the involvement of respective adaptor proteins in CHIKV regulation (49–51). Furthermore, PRR activation and associated innate immune responses in neurons and astrocytes infected with CHIKV remains largely unexplored.

We have previously demonstrated that CHIKV replicates more quickly and to higher titers in astrocytes than neurons; such cell type-dependent dynamics have been linked to the activity of the nsP3^MD^, a neurovirulence-determining factor known to have functions in immune evasion (23, 25, 38, 52, 53). However, how the nsP3^MD^ regulates immunological responses in a cell type-dependent manner is not understood. Therefore, this study first characterized differences in innate immune response to CHIKV infection in neurons versus astrocytes by examining translation and transcription of viral RNA-sensing PRR pathway components and subsequent products such as type I IFN. We further examined how nsP3^MD^ ADPr binding and hydrolase mutations impact expression of type I IFN, and additionally characterized *Parp* transcription, PARP-mediated CHIKV replication dynamics, and global ADP-ribosylation following infection. We demonstrated that, in astrocytes only, CHIKV infection activates RIG-I, as well as subsequent IRF3 phosphorylation and nuclear translocation. In contrast, CHIKV infection in neurons resulted in PARP1 activation and enhanced global ADP-ribosylation. Type I IFN expression was only observed in astrocytes and was found to be influenced by the nsP3^MD^. Together, our findings identify cell type-dependent innate immune responses between astrocytes, or major cerebral immunomodulatory cells, and neurons, or cells known to maintain alphavirus infection, to CHIKV infection.

## MATERIALS AND METHODS

### Cell culture and IFN, Poly I:C Treatment

The murine astrocyte type I clone (C8-D1A, CRL-2541), human astrocytic-like cell (CCF-STTG1, CRL-1718), human neuroblastoma cells (SH-SY5Y, CRL-2266), African green monkey epithelial cells (Vero, CCL-81), baby hamster kidney epithelial cells (BHK-21, CCL-10), and human embryonic kidney epithelial cells (HEK293, CRL-3216) were purchased from American Type Culture Collection (Manassas, VA, USA). The murine neuronal NSC-34 cell line was a kind gift from Dr. Neil Cashman (54). All cells were cultured at 37°C and 5% CO_2_ in Dulbecco’s modified Eagle’s medium (DMEM; Gibco/Thermo Fisher Scientific, Waltham, MA, USA) supplemented with 10% heat–inactivated fetal bovine serum (FBS; Atlanta Biologicals, Flower Branch, GA, USA), L-glutamine (2 mM; Gibco), streptomycin (100 μg/mL; Gibco), and penicillin (100 U/mL; Gibco). All cell lines were documented to be free of mycoplasma (Lonza Bioscience, Walkersville, MD, USA). To mimic double-stranded RNA (dsRNA) activation of innate responses during virus infection, C8-D1A and NSC-34 cells were treated with low-molecular-weight polyinosinic:polycytidylic acid (Poly I:C) at 1 μg/mL (InvivoGen, tlrl-picw; San Diego, CA, USA) combined with Lipofectamine 2000 (Invitrogen/Thermo Fisher Scientific) transfection reagent in DMEM according to the manufacturer’s instructions. For IFN stimulation, cells were treated with 100 IU of Universal Type I IFN (Human IFN–Alpha Hybrid Protein, 11,200, PBL Bioscience, Piscataway, NJ, USA) prepared in DMEM.

### Virus generation, *in vitro* infection, and PARP inhibitor treatment

A full-length complementary DNA (cDNA) clone of the CHIKV 181/25 vaccine strain (55) (a kind gift from Naomi Forrester, University of Texas Medical Branch, Galveston, TX, USA) was used to transcribe viral RNA using mMESSAGE mMACHINE SP6 Kit (Invitrogen/Thermo Fisher Scientific). Mutant viruses harboring G32S or Y144A mutations in the nsP3 MD were made using the CHIKV 181/25 cDNA clone as backbone and and have been previously characterized as attenuated both *in vitro* and *in vivo* (23, 24). Viral stocks were titered by plaque assays using Vero cells. All virus infections were performed in BSL-2 facilities in accordance with Johns Hopkins University institutional biosafety guidelines.

For virus infection, cells were generally infected at a multiplicity of infection (MOI) of 5, unless otherwise specified, incubated in a 37°C and 5% CO_2_ humidified chamber for an hour, and washed once in 1× phosphate-buffered saline (PBS). Media were then replaced with fresh DMEM supplemented with 1% heat inactivated FBS. For PARP inhibitor studies, PARP inhibitors 413A (56) and 413B (57) (provided by Michael S. Cohen, Oregon Health Science University, OR, USA) and the PARP1 inhibitor PJ-34 (HY-13688A) and Olaparib (HY-10162) (both from MedChemExpress, Monmouth Junction, NJ, USA) or ABT-888 (Selleck Chemicals, S1004; Houston, TX, USA) were added to the media 1 h post-infection (hpi) at an MOI 1. Cell viability was measured using CellTiter 96 Aqueous One Solution Cell Proliferation Assay (Promega, Madison, WI, USA), as per manufacturer’s instruction.

### IFNα and IFNβ ELISAs

Supernatant from C8-D1A, CCF-STTG1, and SH-SY5Y cells mock or infected with CHIKV 181/25 (wild-type [WT]) or CHIKV nsP3^MD^ mutants at an MOI of 5 were collected at different hpi. Concentrations of IFNα and IFNβ were measured using VeriKine ELISA kits (PBL Assay Science; 42120, 42400, 41435, 41135; Piscataway, NJ, USA), according to the manufacturer’s instructions. Supernatants from three independent experiments were tested. Assay ranges were 12.5–400 pg/mL for mouse IFNα, 15.6–1,000 pg/mL for mouse IFNβ, 1.95–125 pg/mL for human IFNα, and 2.34–150 pg/mL for human IFNβ ELISA kits. The limit of detection is considered half the value of the lowest assay range.

### RT-qPCR for genes encoding PARPs, type I IFN, RLRs, and TLRs

C8-D1A, SH-SY5Y, and NSC-34 cells were infected with mock or CHIKV 181/25 (WT) at an MOI of 5. NSC-34 cells were additionally either mock-treated or treated with 100 IU of Universal Type I IFN; lysates were then harvested in RLT buffer (Qiagen, Hilden, Germany) at different hpi. Total RNA was collected and isolated using the RNeasy Plus Mini Kit (Qiagen); cDNA was synthesized using the High-Capacity cDNA Reverse Transcription Kit (Applied Biosystems/Fisher Scientific, Waltham, MA, USA). TaqMan gene expression arrays or gene specific primer set (Integrated DNA Technologies, Coralville, IA, USA) was used to measure genes encoding murine and human PARPs, as well as other innate immune response genes such as *Irf3*, *Irf7*, *Ifna*, *Ifnb*, *Rigi*, *Mda5*, *Tlr3*, *Tlr7*, *Tlr9*, *Myd88*, *Cgas*, and *Sting* mRNA levels. Mouse *Gapdh* was quantified using a commercial kit (Applied Biosystems/Fisher Scientific; 4308313). RT-qPCR was performed using Universal PCR Master Mix or SYBR Green Master Mix (Applied Biosystems/Fisher Scientific). The qPCR conditions consisted of 40 cycles of 2 min at 50°C, 10 min at 95°C, and 1 min at 60°C performed in a 7500 Real-time PCR machine (Applied Biosystems/Fisher Scientific). Relative gene expression was determined using the ΔΔCt method using 0-h control samples, as well as *Gapdh/GAPDH* or *Rps29* for normalization. The primers used are delineated in Table 1.

**TABLE 1:** List of primers used for the study

List of primers		
Species	Primer	Cat. no. or sequence (5′ to 3′)
Mouse	*Gapdh*	43-083-13
Mouse	*Parp1*	Mm.PT.58.16816351
Mouse	*Parp2*	MM.PT.58.7099437
Mouse	*Parp4*	Mm.PT.58.28919965
Mouse	*Parp7*	Mm.PT.58.31595971
Mouse	*Parp9*	Mm.PT.58.11565829
Mouse	*Parp10*	Mm.PT.58.12367998
Mouse	*Parp11*	Mm.PT.58.6353501
Mouse	*Parp12*	Mm.PT.58.7540423
Mouse	*Parp13*	Mm.PT.58.31085338
Mouse	*Parp14*	Mm.PT.58.45872106
Mouse	*Rigi*	Mm.PT.58.9774198
Mouse	*Mda5*	Mm.PT.58.28930855
Mouse	*Mavs*	Mm.PT.58.28896835
Mouse	*Tlr3*	Mm.PT.58.8085919
Mouse	*Myd88*	Mm.PT.58.33389595
Mouse	*Tlr7*	Mm.PT.58.10526075
Mouse	*Tlr9*	Mm.PT.58.5114450
Mouse	*Sting*	Mm.PT.58.12798185
Mouse	*cGas F*	ACGAGAGCCGTTTTATCTCGTACCC
Mouse	*cGas R*	TGTCCGGAAGATTCACAGCATGTTT
Mouse	*Ifna*	Mm.PT.58.43426930.g
Mouse	*Ifnb*	Mm.PT.58.30132453.g
Mouse	*Irf3*	Mm.PT.58.5650168
Mouse	*Irf7*	Mm.PT.58.32394021.g
Mouse	*Rps29* F	AGCAGCTCTACTGGAGTCACC
Mouse	*Rps29* R	AGGTCGCTTAGTCCAACTTAATG
Human	*PARP1 F*	TGAGGTCCAGCAGGCGGTGT
Human	*PARP1 R*	AGTCGTGGGGGATCAGGGTGT
Human	*PARP9 F*	TGGCGCTCGTTAGGACAGTGG
Human	*PARP9 R*	GCTCTTGAGTTGGAGGCACAGGA
Human	*PARP10 F*	GCACCGGCAGTGCCTGACAT
Human	*PARP10 R*	CCCGTAGACCGTGGCGTTGC
Human	*PARP11 F*	ACGTCAGATACCCAGTGGGGCTG
Human	*PARP11 R*	TGGTATCCGGCTGAAACATGTGC
Human	*PARP12 F*	CCTACGGCAAGGGGAGCTACTT
Human	*PARP12 R*	TCGTGTGGGTCTGCGTGTCG
Human	*PARP13.1 F*	GAGAGGGCCAGACCATCAGCCA
Human	*PARP13.1 R*	CCTCCTGAGGACGAAAGGTCGCA
Human	*PARP13.2 F*	GCAGCAGATGAAGAGAGGGCCA
Human	*PARP13.2 R*	TGAGCCCAGGGCATGAACATCT
Human	*PARP14 F*	GGCTCCGTGCCACACGTCAA
Human	*PARP14 R*	CATATGCCACAGCATTCTTTCCGGC
Human	*PARP15 F*	TCGGAAATCAGGTGTGTCAAGAGC
Human	*PARP15 R*	TCTGTGAGGCTGTGCAGCTAGT
Human	*PARP16 F*	AACCAGCTGCTGCGAGTGAAGT
Human	*PARP16 R*	GGCTCGAAGCCCTGCTCTTGG
Human	*GAPDH F*	CCATCACTGCCACCCAGAAGAC
Human	*GAPDH R*	GGCAGGTTTTTCTAGACGGCAG

### Western blot analysis of protein expression

Cells were mock- or CHIKV WT-infected at an MOI of 5, according to previously described protocols, and incubated at 37°C. At selected time points, the cells were lysed in RIPA buffer (50 mM Tris [pH 8], 1% Triton X-100, 0.1% of SDS, 150 mM NaCl, 1 mM EDTA, and 0.5% Na_3_VO_4_2H_2_O), supplemented with complete protease and phosphatase inhibitors (Roche, Basel, Switzerland), on ice for 30 min followed by centrifuging at 15,200 × *g* for 10 min at 4°C. The DC protein assay (Bio-Rad, Hercules, CA, USA) was utilized to estimate total protein in lysates with bovine serum albumin (BSA) as the standard. Following protein normalization and linearization via heat treatment, a total of 15 to 20 µg of protein per sample was loaded onto a 10% SDS-PAGE gel, which was separated via electrophoresis and transferred to nitrocellulose membranes. The membranes were blocked with 5% milk in TBS supplemented with 1% Tween-20 (TBS-T) at room temperature (RT), followed by overnight incubation at 4°C with antibodies specific to pan-ADP-ribose (Millipore/Sigma-Aldrich), RIG-I, MAVS, phospho-IRF3, IRF3, phospho-IRF7, IRF-7, PARP1, Caspase 3 (1:1,000; Cell Signaling Technologies, Danvers, MA, USA), MDA5, (1:500; Abcam, Cambridge, UK), nsP3 (1:1,000; a kind gift from Dr. Andres Merits (University of Tartu) or β-actin (1:5,000; Millipore/Sigma-Aldrich) diluted in 5% BSA in TBS-T. Secondary antibodies comprised HRP-conjugated anti-mouse and HRP-conjugated anti-rabbit (1:1,000; Cell Signaling Technologies). Secondary antibodies were diluted in 2% milk prepared in TBS-T and incubated for an hour at RT. Amersham ECL Prime Western Blotting Detection Reagent (Cytiva, Marlborough, MA, USA) was used to develop the membrane according to manufacturer protocols. ImageJ software from the NIH was used to analyze the intensity of bands and densitometry of immunoblots from three independent experiments.

### Cytoplasmic and nuclear fractionation

Mock or CHIKV WT-infected C8-D1A and NSC-34 cells were trypsinized at designated hpi and transferred to clean 1.5-mL tubes. Following PBS washing of cell pellets, nuclear and cytoplasmic fractionation was performed using an NE-PER Nuclear Cytoplasmic Extraction Reagents Kit (Thermo Fisher Scientific; 78833) according to the manufacturer’s protocols, but with three additional washing steps of the pellet containing nuclear material. Once fractions were collected and protein has been quantified, samples were further processed using 6× SDS cracking buffer and subsequently analyzed via Western blotting.

### Co-immunoprecipitation of nsP3

Confluent, low-passage C8-D1A and NSC-34 cells were mock- or CHIKV WT-infected at an MOI of 5. At 12, 24, and 36 hpi, cells were washed once using PBS and then lysed using 100 µL of an in-house RIPA co-IP lysis buffer (50 mM Tris pH 8, 150 mM NaCl, 1% NP40, 5% Na deoxycholate, 1 mM EDTA) supplemented with cOmplete Mini Protease Inhibitor Cocktail (Roche, Cat. No. 11836153001). RIPA co-IP lysis buffer (“input”), rabbit IgG isotype control (GeneTex, Irvine, CA, USA; GTX35035; “Negative”), or CHIKV nsP3 polyclonal antibody (GeneTex, GTX135189; “Output”) was added to designated lysates at a final concentration of 1:100 and incubated overnight at 4°C on a rotating shaker. Pierce Protein A/G Agarose beads were then added to designated samples (“Negative” and “Output”) according to manufacturer recommendations; samples were incubated for 5 h at RT and subsequently washed thrice using 500 µL of RIPA co-IP lysis buffer. All samples were then incubated with 6× SDS cracking buffer and analyzed via Western blot analysis, as described in aforementioned protocols.

### Chemical synthesis and PASTA

For the synthesis of 413A see Supplemental material. Synthesis of 413B (Pthal01) was previously described (57). PARP inhibitor family-wide screening (PASTA) was performed as previously described (58, 59) Inhibitor dose-response curves were fitted using linear regression. The mean IC_50_ for each compound was calculated from at least three independent assays.

### Lentivirus transduction and stable cell line generation

HEK293T cells were transfected with the following plasmid cocktail to generate lentiviral particles: lentiviral small hairpin RNA (shRNA) construct (either shPARP1 [TRCN0000305949; NM_007415] or control TRC2 pLKO.5 puro [Millipore/Sigma-Aldrich, St. Louis, MO, USA]), psPAX2 and pMD2.G (both from Addgene, Watertown, MA, USA) at a ratio of 3:2:1, respectively. Transfection was performed using Lipofectamine 2000 according to the manufacturer’s instructions. At 48 h post-transfection, the supernatant was collected, centrifuged, and filtered through a 0.45-μm low-protein-binding filter. The clarified virus suspension containing lentiviral particles was transduced into NSC-34 cells overnight. Fresh media containing puromycin (Millipore/Sigma-Aldrich; P-9620) at 2 μg/mL final concentration were added for selection, and single cell colonies were picked for shRNA-based genetic depletion of PARP1 in NSC-34 cells.

### Statistical analysis

Time course studies were analyzed using two-way analysis of variance (ANOVA) with Tukey’s multiple-comparison test to compare the groups. Differences in a single group were determined using one-way ANOVA with Dunnett’s multiple-comparison test. Differences between groups at a single time point were determined using an unpaired two-tailed Student’s t-test with a 95% CI. The results are expressed as means ± standard deviation (SD) from three independent biological experiments. Statistical analyses were conducted using GraphPad Prism 8 Software.

## RESULTS

### Retinoic acid inducible gene-I-like receptors are activated during chikungunya virus infection in astrocytes, but not neurons

**Fig 1:**
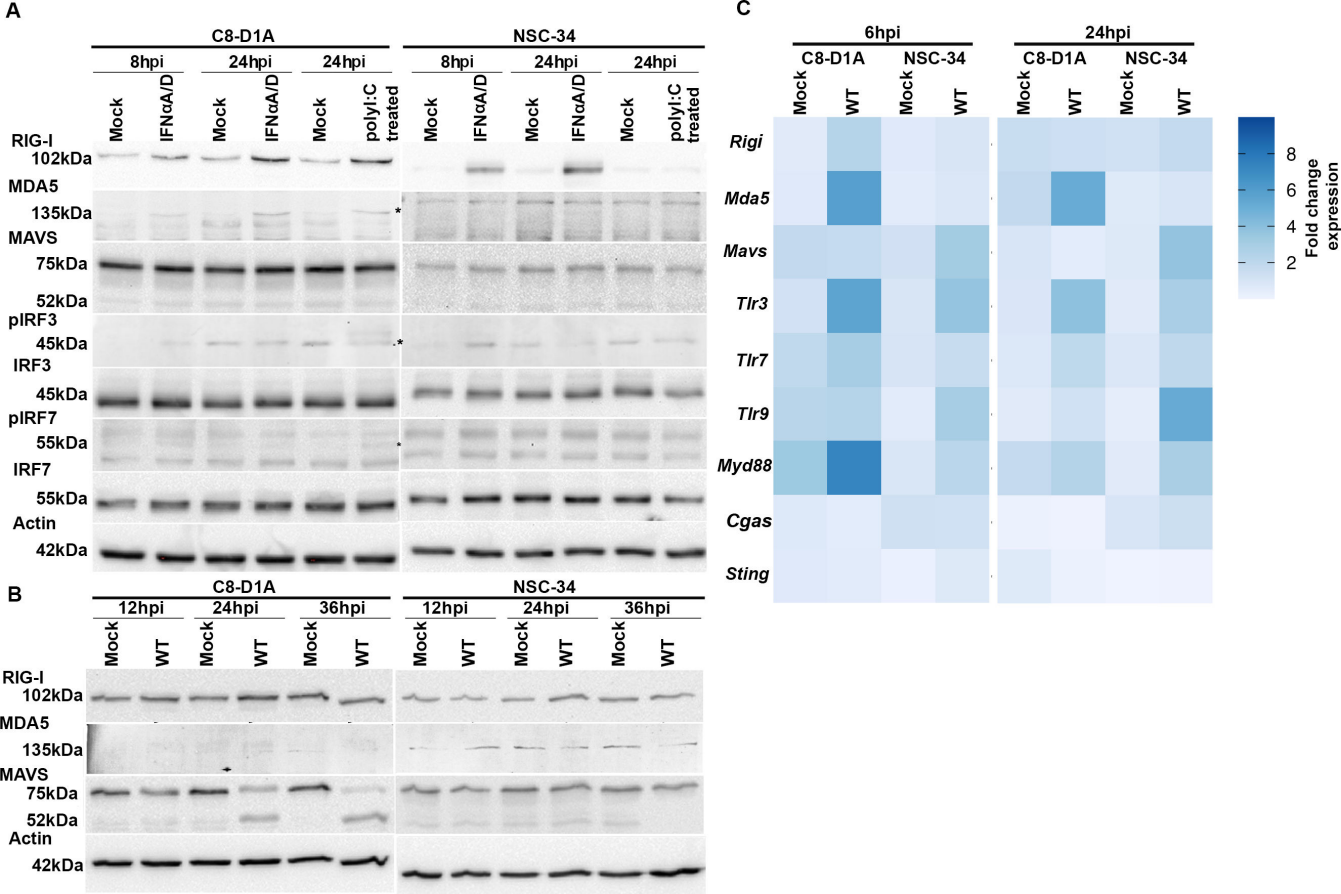
RIG-I-like receptor pathway activation in astrocytes and neurons. (**A**) To activate antiviral PRRs related to type I IFN, C8-D1A and NSC-34 cells were treated with 100 U of universal type I IFNα A/D or transfected with 1 μg/mL of low-molecular-weight poly(I:C). Twenty-four hours after treatment, lysates (*n* = 3 biological samples per time point, one biological sample per Western blot) were collected, and immunoblots were prepared to determine RLR pathway receptor and mediator expression. A representative image from three biological experiments is presented. (**B**) Lysates from mock-infected or WT CHIKV-infected (MOI 5) C8-D1A cells and NSC-34 cells were immunoblotted for RIG-I, MDA5, MAVS, and β-actin, as demonstrated by a representative image from three biological experiments. (**C**) RT-qPCR of RNA isolated from mock- and WT CHIKV-infected NSC-34 and C8-D1A cells (MOI 5) (*n* = 3 biological samples per time point) was performed to quantify relative gene expression, as normalized to *Gapdh*. Fold change of expression was calculated by comparison to the 0-h mock-infected samples. A heat map was generated for the 6 and 24 hpi time-points for both cell lines. Corresponding graphs for each gene across the full-time course are provided in Fig. S1, along with appropriate statistical analyses.

CHIKV replicated more quickly in C8-D1A murine astrocytes and to higher titers than in NSC-34 neuronal cells; such cell type-specific CHIKV replication dynamics is directly linked to the function of the nsP3^MD^ (38, 52), which possesses immune evasive functions (23, 25, 53). However, how the nsP3^MD^ regulates immunological responses in these two different cell types remains unknown. To investigate differences in immune responses to infection between these two cell types, we characterized RNA PRRs and downstream innate immune signaling pathways in CHIKV-infected astrocytes versus neurons. To establish baseline, cell type-specific RLR responses, cells were stimulated with poly(I:C), a synthetic analog of dsRNA that mimics RNA intermediates produced during viral replication. The expression of RLRs and their downstream mediators was assessed by immunoblotting (Fig. 1A). When treated with poly(I:C), C8-D1A murine astrocytes showed increased expression of RIG-I and MDA5, while mitochondrial antiviral signaling protein (MAVS) levels, a key adaptor protein of RIG-I and MDA5 RLRs, remained unchanged relative to mock-treated. In contrast, poly(I:C) treatment of NSC-34 murine neurons did not activate these tested components of the RLR signaling pathway (Fig. 1A). We further examined baseline neuronal versus astrocyte RLR responses to exogenous type I IFN via universal type I IFNα A/D treatment. Type I IFN treatment resulted in RIG-I activation in both cell lines, whereas MDA5 was activated only in the astrocytic line. No changes in MAVS were observed in either neurons or astrocytes. Finally, we examined RLR responses in both cell lines to CHIKV infection. In infected neurons and astrocytes, RIG-I receptors appear to be constitutively expressed in both astrocytes and neurons, regardless of infection status (Fig. 1B). However, CHIKV infection resulted in increased RIG-I band signal intensity relative to mock in astrocytes, but not neurons (Fig. 1B). No detectable band for MDA5 was observed in C8-D1A cells, and no modulation of MDA5 levels was noted upon CHIKV infection in NSC-34 cells (Fig. 1B). Interestingly, MAVS activation was apparent at 24 and 36 hpi in astrocytes, but not in neurons, as evidenced by the appearance of a 52 kDa cleaved product from the parental 75 kDa band (Fig. 1B). Therefore, dsRNA or type I IFN treatment activates RIG-I and MDA5 expression in astrocytes, while CHIKV infection activates only RIG-I expression in astrocytes. Neither CHIKV infection nor exogenous type I IFN or dsRNA treatment activates RLR components in NSC-34 neurons. Although the levels of pIRF7 and total IRF7 did not change with any treatment in either astrocytes or neurons, pIRF3 levels were altered following exogenous type I IFN treatment at early time points.

To further investigate the expression of major RNA PRRs in astrocytes versus neurons at the mRNA level during CHIKV infection, C8-D1A astrocytes and NSC-34 neurons were infected with WT CHIKV. Total RNA was isolated from cell lysates at different hpi and subjected to RT-qPCR analysis to assess transcript levels of major RNA PRRs such as *Rigi*, *Mda5*, *Tlr3*, and *Tlr7*. DNA-sensing PRRs such as *Tlr9*, *Cgas*, and *Sting* were also included, considering their importance to CHIKV infection disruption. A heat map derived from the RT-qPCR data at 6 and 24 hpi shows significant early and late C8-D1A astrocytic upregulation of *Rigi* (6 hpi, *P* < 0.0001; 24 hpi*, P* < 0.001) and *Mda5* (6 hpi, *P* < 0.0001; 24 hpi, *P* < 0.001), both associated with the RLR pathway (Fig. 1C; Fig. S1). In contrast, *Rigi* and *Mda5* expression showed no significant upregulation until 36 hpi (*P* < 0.05; Fig. 1C; Fig. S1). No statistically significant upregulation of *Tlr3*, *Tlr7*, or *Tlr9* gene expression was observed in either C8-D1A or NSC-34 cells. Interestingly, *Myd88*, which encodes the adaptor protein associated with TLR7 and TLR9 signaling, was significantly upregulated at an early phase (6 hpi, *P* < 0.0001). Although CHIKV is known to degrade cGAS and antagonize cGAS–STING-mediated type I IFN responses, neither *Cgas* nor *Sting* showed significant modulation in either murine cell line upon infection (Fig. 1; Fig. S1) (60). We have thus far demonstrated *Rigi* and *Mda5* upregulation in CHIKV-infected astrocytes, but not neurons. Therefore, we focused further on downstream components of the RIG-I pathway.

### Type I interferon is expressed in astrocytes, but not neurons, during chikungunya virus infection and is influenced by the non-structural protein 3 macrodomain

**Fig 2:**
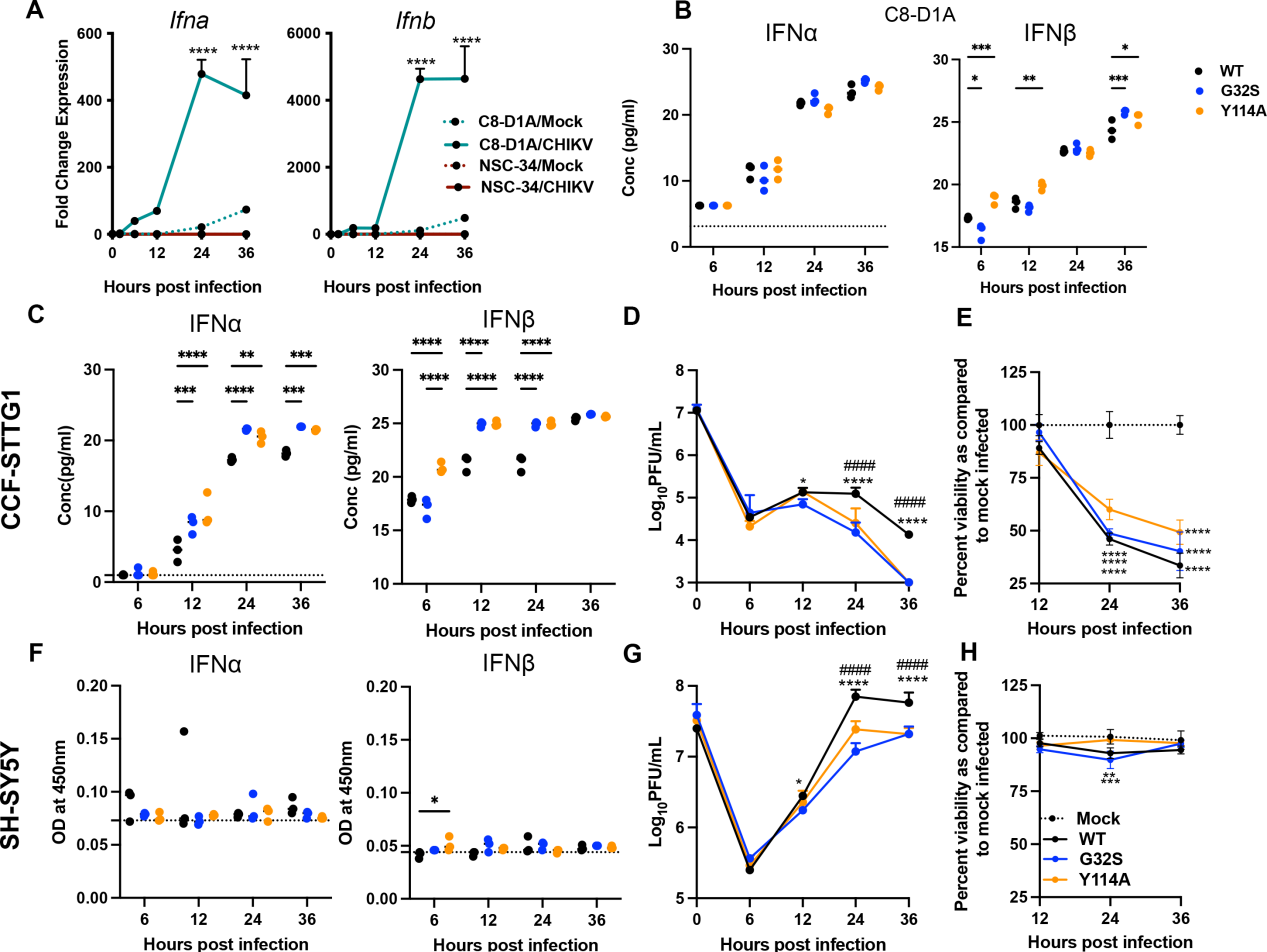
Type I IFN response by CHIKV-infected astrocytes and neurons. (**A**) NSC-34 and C8-D1A cells were either mock-infected or infected with WT CHIKV (MOI 5); RNA was subsequently isolated (*n* = 3 biological samples per time point) and cDNA was synthesized. RT-qPCR was performed to quantify relative *Ifnɑ* and *Ifnb* gene expression. The C_t_ value for each transcript was normalized to *Gapdh.* Fold change in expression was calculated relative to 0-h mock-infected samples. Data represent the average ± SD. ****P < 0.0001 (C8-D1A CHIKV vs NSC-34 CHIKV). Supernatants of (**B**) C8-D1A , (**C**) CCF-STTG1, (**F**) SH-SY5Y cells infected with CHIKV WT or nsP3^MD^ mutants were collected (MOI 5) (*n* = 3 biological samples per time point) and type I IFN was measured by ELISA. Data represent the average ± SD. ****P < 0.0001, ***P < 0.001, **P < 0.01, *P < 0.05 (WT vs G32S/Y114A). Supernatants of (**D**) CCF-STTG1 or (**G**) SH-SY5Y cells infected with CHIKV WT or nsP3 MD mutants (MOI 5) were collected (*n* = 3 biological samples per time point), and virus titration was performed by plaque assays using Vero cells. Data represent the average ± SD. *P < 0.05, ****P < 0.0001 (WT vs G32S), ^####^*P <* 0.0001 (WT vs Y114A). (**E**) CCF-STTG1 and (**H**) SH-SY5Y cells were infected with CHIKV WT or nsP3 MD mutants (MOI 5) and, at corresponding hpi, CellTiter-Glo reagent was added and cell viability was measured per manufacturer’s instruction (*n* = 5 biological samples per time point). Data represent the average ± SD. ****P < 0.0001, ***P < 0.001, **P < 0.01 (mock vs WT/nsP3 MD mutants).

We further investigated the expression of type I IFN, a cytokine known to be activated by RIG-I expression, in CHIKV-infected neurons and astrocytes. We found significant upregulation of *Ifna* and *Ifnb* in CHIKV-infected C8-D1A astrocytes, but not in NSC-34 neurons, with peak *Ifna* and *Ifnb* transcripts observed at 24 and 36 hpi in astrocytes (*P* < 0.0001; Fig. 2A). No *Ifna* or *Ifnb* expression was observed in NSC-34 neurons, as expected (24). Previous SARS-CoV-2 studies have demonstrated nsP3^MD^-mediated impairment of type I IFN expression, as well as reversal of PARP14-mediated MARylation associated with IFN pathway induction (33, 61). Inactivation of MD properties such as ADPr binding and removal furthermore attenuates viral fitness while simultaneously restoring host IFN responses (23, 25, 61–63). Thus, considering our previous work demonstrating cell type-dependent effects of the nsP3^MD^ on astrocytic versus neuronal replication dynamics, as well as the divergence in baseline *Ifna* and *Ifnb* transcript levels in both cell types, we postulated that the nsP3^MD^ may play a role in modulating type I IFN pathways in a cell type-specific manner. We therefore performed enzyme-linked immunosorbent assays (ELISAs) to quantify type I IFN protein levels in murine astrocytes upon infection with CHIKV WT or nsP3^MD^ mutants (Fig. 2B). Furthermore, the inclusion of MD mutants G32S (low ADPr binding and hydrolase capacity) and Y114A (higher ADPr binding, but low hydrolase capacity) allowed us to further compare cell type-specific differences in nsP3^MD^ mutant virus replication dynamics, as Y114A mutation attenuates viral replication in neurons, but not astrocytes (24, 38), while G32S mutation hinders replication in both cell types, relative to WT. We have previously demonstrated that neither IFNα nor IFNβ are produced upon CHIKV infection of NSC-34 neurons (24). In this study, we found that, in WT-infected C8-D1A cells, IFNα and IFNβ were detected as soon as 12 and 6 hpi, respectively (Fig. 2B). No differences were observed in IFNα levels between WT and nsP3^MD^ mutant-infected cells (Fig. 2B). IFNβ production was higher in Y114A-infected C8-D1A cells at earlier time points (*P* < 0.001, 6 hpi; *P* < 0.01, 12hpi) and later time points in both mutants (*P* < 0.001, G32S; *P* < 0.05, Y114A) compared to WT virus infection.

To further examine type I IFN production in other astrocytic cell lines, we used a human astrocyte cell line, CCF-STTG1, and a human neuronal cell line, SH-SY5Y. Type I IFN expression dynamics of CCF-STTG1 mimicked that of the C8-D1A cells (Fig. 2C); however, Y114A and G32S mutants showcased significantly higher IFNα levels than WT (*P* < 0.001, 12/36 hpi; *P* < 0.0001, 36 hpi, G32S; *P* < 0.0001, 12 hpi; *P* < 0.01, 24 hpi; *P* < 0.001, 36 hpi, Y114A) and IFNβ (*P* < 0.0001, 12/24/36 hpi, G32S/Y114A). Interestingly, type I IFN production in CCF-STTG1 astrocytes appeared to inversely correspond with infectious virus titers (Fig. 2D), with higher IFNα and IFNβ expression associated with lower viral replication. CCF-STTG1 cells were highly susceptible to CHIKV infection, resulting in pronounced cytopathic effects and low viral titers (Fig. 2E). Similar to NSC-34 cells, type I IFNs were not detectable in SH-SY5Y neuronal cells (Fig. 2F), despite efficient CHIKV replication and minimal cytopathic effect, as compared with CCF-STTG1 cells (Fig. 2G and H). We found that both IFNα and IFNβ expression were significantly upregulated in CHIKV-infected astrocytes, but not neurons. Furthermore, the nsP3^MD^ regulated IFNα and IFNβ production in human astrocytes, with higher levels of type I IFN corresponding with decreased viral replication.

### CHIKV infection in astrocytes, but not neurons, induces IRF3 phosphorylation and subsequent nuclear translocation

**Fig 3:**
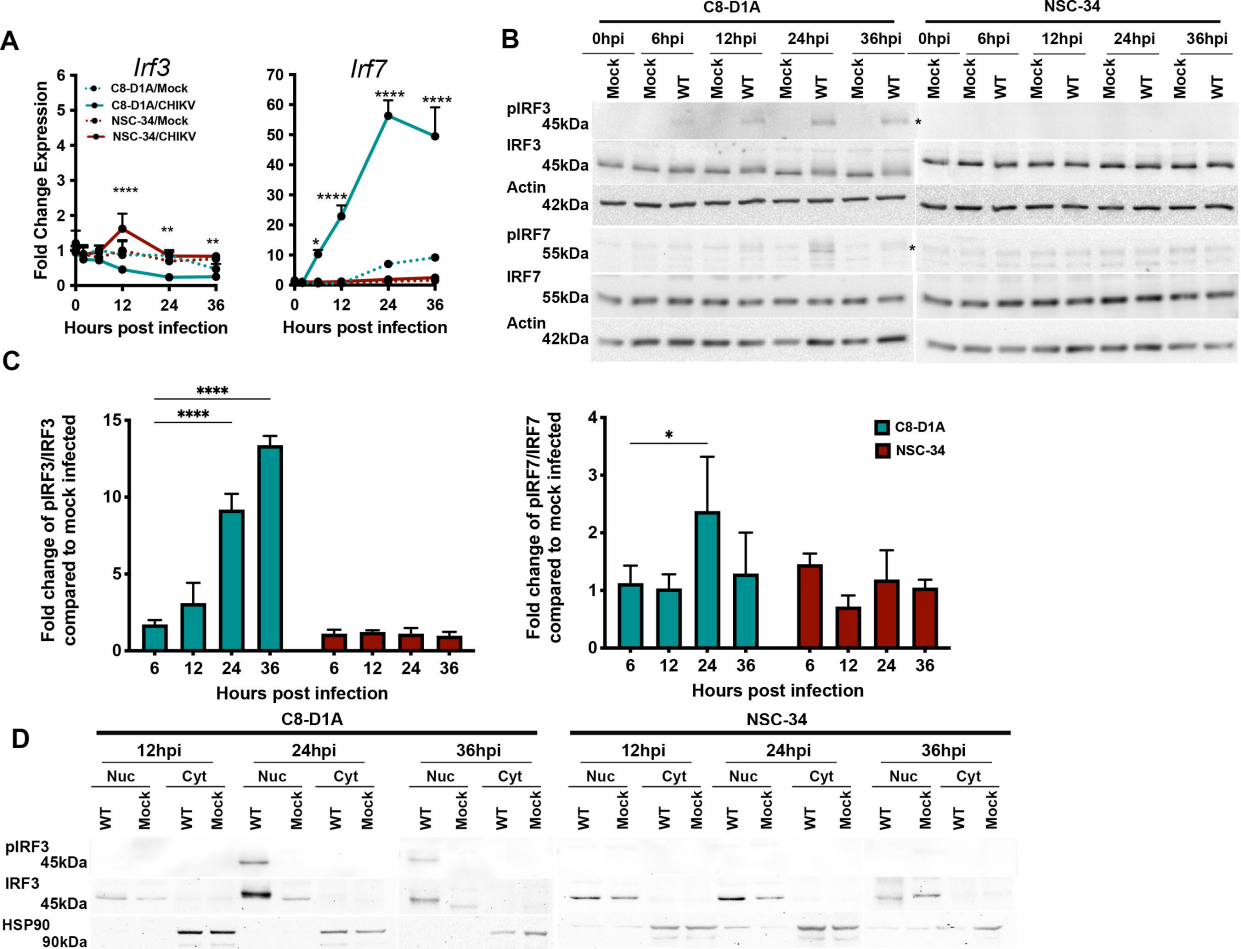
Phosphorylation of IRF3 and its nuclear translocation in chikungunya virus-infected astrocytes. (**A**) NSC-34 and C8-D1A cells were either mock-infected or infected with WT CHIKV (MOI 5); RNA was then isolated (*n* = 3 biological samples per time point) and cDNA synthesized. RT-qPCR was performed for *Irf3* and *Irf7* to determine changes in gene expression. C_t_ values for each transcript was normalized to *Gapdh.* Fold change in expression was calculated relative to 0-h mock-infected samples. Data represent the average ± SD. ****P < 0.0001, **P < 0.01, *P < 0.05 (C8-D1A CHIKV vs NSC-34 CHIKV). (**B**) Lysates from C8-D1A cells and NSC-34 cells either mock-infected or infected with WT CHIKV (MOI 5) were immunoblotted for phosphorylated IRF3 (pIRF3), IRF3, phosphorylated IRF7 (pIRF7), IRF7, and β-actin. A representative image from three biological experiments is presented. (**C**) The ratio of pIRF3 to total IRF3 or IRF7 to pIRF7 from 3 to 4 blots was quantified by densitometry and normalized to β-actin. ****P < 0.0001 (6 vs 24/36 hpi, pIRF3/IRF3), *P < 0.05 (6 vs 24 hpi, pIRF7/IRF7). (**D**) Nuclear and cytoplasmic fractions from C8-D1A cells and NSC-34 cells either mock-infected or infected with WT CHIKV (MOI 5) were immunoblotted for pIRF3, IRF3, and Hsp90. A representative image from three biological experiments is presented.

We examined expression of IRF3 and IRF7, key transcription factors downstream of RIG-I signaling that drive type I IFN responses to viral infection. Immunoblotting of CHIKV-infected or mock-infected C8-D1A astrocytes and NSC-34 neurons demonstrated phosphorylation of IRF3, but not IRF7, in astrocytes, but not neurons, following poly(I:C) treatment (Fig. 1A). IFNα A/D treatment did not modify IRF3 or IRF7 expression or phosphorylation in either cell line. Upon CHIKV infection, *Irf3* mRNA was only upregulated in infected neurons, but not astrocytes (*P* < 0.0001, 12 hpi; *P* < 0.01, 24 and 36 hpi; Fig. 3A). *Irf7* expression, on the other hand, increased only in astrocytes from 12 hpi onwards (*P* < 0.0001), as compared with mock-infected cells (Fig. 3A). When examining protein, CHIKV infection did not modify IRF3 expression; however, infection increased IRF3 molecular weight (MW), of which can be attributed to increased IRF3 phosphorylation (pIRF3; Fig. 3B and C). Neither a change in MW nor the appearance of pIRF3 was observed in the neuronal cell line NSC-34 (Fig. 3B and C). Phosphorylation of IRF7 was undetectable until 12 hpi in astrocytes; however, by 24 hpi, C8-D1A cells exhibited significantly more phosphorylated IRF7 than mock control (*P* < 0.01; Fig. 3B and C). No change in pIRF7 expression was observed between CHIKV-infected and mock-infected neurons. To further investigate cell type-specific differences to CHIKV infection, we examined pIRF3 expression by separating nuclear and cytoplasmic protein fractions collected from CHIKV- and mock-infected astrocytes and neurons, and probed for IRF3, pIRF3, and Hsp90 (Fig. 3D). We observed an upward mobility shift of IRF3 in astrocytes following CHIKV infection, suggesting post-translational modification. We further observed pIRF3 nuclear translocation in astrocytes at 24 and 36 hpi, which corresponds to the increase in IRF3 MW. No IRF3 MW changes were observed in mock-infected or CHIKV-infected neurons. Furthermore, no pIRF3 expression in neurons in either cytoplasmic or nuclear compartments was observed. CHIKV infection therefore results in IRF3 phosphorylation and nuclear translocation in astrocytes but not neurons.

### CHIKV infection of neurons, but not astrocytes, induces ADP-ribosylation

**Fig 4:**
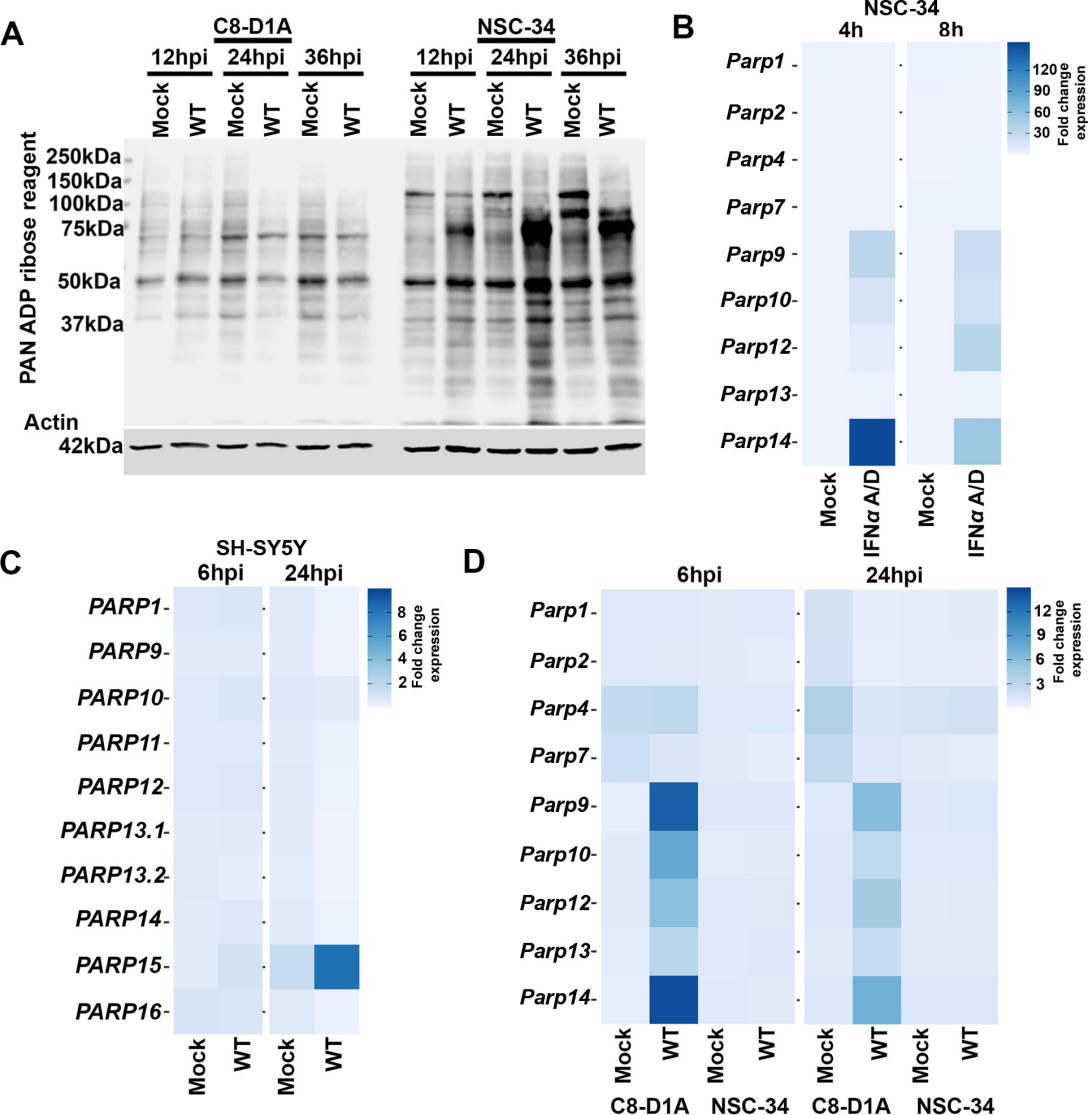
ADP-ribosylation and PARP modulation in astrocytes and neurons. C8-D1A and NSC-34 cells were either mock-infected or infected with WT CHIKV at an MOI 5. (**A**) Lysates were immunoblotted for pan-ADP-ribose reagent and β-actin. A representative image from three biological experiments is shown. (**B**) NSC-34 cells were treated or mock-treated with 100 U of IFNα A/D, RNA was isolated (*n* = 3 biological samples per time-point), and cDNA was synthesized. Gene expression was determined by real-time PCR; C_t_ values were normalized to *Gapdh.* Fold change of expression was calculated by comparison to the 0-h mock-treated samples. A heat map of gene expression at 4 and 8 h post-treatment is presented, with corresponding graphs and appropriate statistical analyses for each gene across the full-time course provided in Fig. S3B. (**C**) SH-SY5Y, (**D**) C8-D1A and NSC-34 cells were either mock-infected or infected with CHIKV WT (MOI 5), RNA was isolated (*n* = 3 biological samples per time point), cDNA was synthesized, and gene expression was determined as mentioned above. Fold change of expression was calculated through comparison to the 0-h mock-infected samples. The resulting heat map was generated from 6 and 24 hpi. Data show the mean ± SD. Corresponding graphs for each gene across the full-time course are provided in Fig. S3C and D, along with appropriate statistical analyses.

Since we observed type I IFN induction in response to CHIKV infection in astrocytes, but not neurons, further elucidation of cell type-dependent effects of type I IFN stimulation is necessary to understand CHIKV interactions in both cell types. ADP-ribosylation events are mediated by the activation of PARPs, most of which are induced by type I IFNs. Therefore, to understand how host ADP-ribosylation induction to infection differs in astrocytes and neurons, cell lysates were collected at various time points post-infection and subjected to Western blot analysis using the pan-ADPr-reagent, which detects both MARylated and PARylated substrates. Unlike the robust ADP-ribosylation observed in NSC-34 neuronal cells following CHIKV infection (24), C8-D1A astrocytes showed either a modest reduction or no significant change in total ADP-ribosylation relative to mock-infected controls (Fig. 4A). Consistently, H₂O₂-induced oxidative stress elicited substantially lower levels of ADP-ribosylation in astrocytes than in neuronal cells (Fig. S2A), suggesting that ADP-ribosylation responses are cell-type dependent. However, we previously observed that CHIKV infection in NSC-34 neurons did not modulate *Parp* transcripts encoding type I IFN-stimulated PARPs, as expected (Fig. 4B; Fig. S2B) (24). To determine whether this observation is consistent across other neuronal cells, we infected human SH-SY5Y neurons with CHIKV WT and assessed *PARP* levels (Fig. 4C; Fig. S2C). Most *PARP* transcripts, including *PARP1*, *PARP9*, *PARP10*, *PARP11*, *PARP12*, *PARP13.1*, *PARP13.2*, *PARP14*, and *PARP16*, were not modulated in comparison to mock-infected cells. Notably, *PARP15*, which lacks a murine homolog and encodes a MAR-adding ART, was upregulated in CHIKV-infected SH-SY5Y cells starting at 12 hpi (*P* < 0.0001) and peaked at 36 hpi (*P* < 0.0001; Fig. 4C). In CHIKV-infected C8-D1A murine astrocytes, genes encoding type I IFN-responsive PARPs, such as *Parp9*, *Parp10*, *Parp13*, and *Parp14*, were upregulated, with transcript levels peaking at 6 hpi (*P* < 0.0001 for *Parp9, Parp10*, *Parp13; P* < 0.001 for *Parp14*), while *Parp12* peaked at 12 hpi (*P* < 0.0001; Fig. 4D; Fig. S2D). Other PARP genes like *Parp1*, *Parp2*, *Parp4*, and *Parp7* were not modulated during the course of infection. Such differential expression of PARP genes in astrocytes and neurons suggests that the cellular response to infection is cell type-specific, as astrocytes upregulate *Parp* transcription without modifying ADP-ribosylation activity, but neurons express increased total ADP-ribosylation upon CHIKV infection without showcasing changes in *Parp* transcript levels.

**Fig 5:**
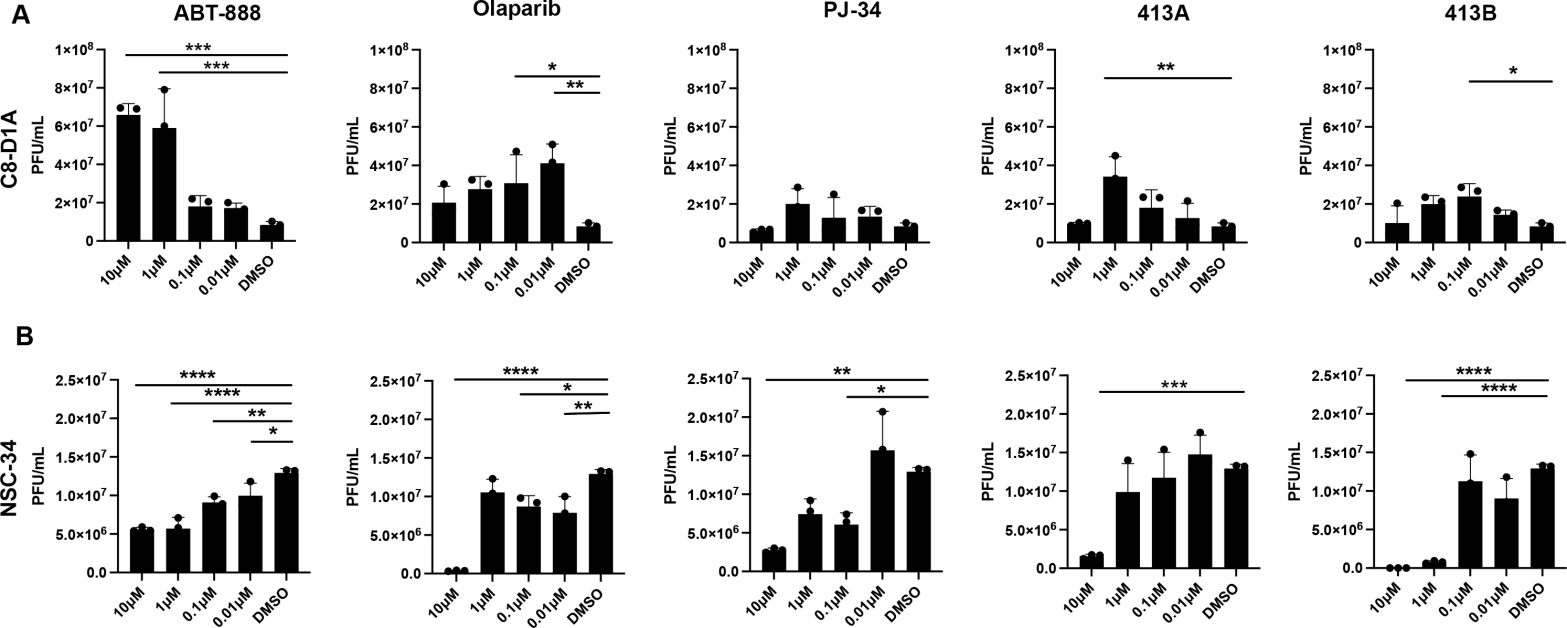
CHIKV replication in astrocytes and neurons following treatment with specific PARP inhibitors. (**A**) C8-D1A and (**B**) NSC-34 cells were either mock-infected or infected with WT CHIKV at an MOI of 1 and treated with 0.1–10 μM of PARP1- and PARP2-specific inhibitors ABT-888, Olaparib, and PJ-34, or broad PARP inhibitors 413A and 413B for 24 hpi. Supernatants were collected, and Vero plaque assay were performed. Data represent the average ± SD. *P < 0.05, **P < 0.01, ***P < 0.001, ****P < 0.0001 in comparison with DMSO.

To determine which ARTs are essential during CHIKV replication in both astrocytes and neurons, we treated the cells post-CHIKV infection using the following PARP inhibitors: 413A, targeting PARPs 1-6 (56); 413B, targeting all PARPs (57); or ABT-888, PJ-34, or Olaparib, all of which potently inhibit PARP1 and PARP2 (64). The chemistry for 413A synthesis is described in Supplementary files. The PARP selectivity profiles of inhibitors 413A and 413B are summarized in Table S1. C8-D1A and NSC-34 cells were treated with 0.1–10 μM of each drug for 24 h immediately following infection. Vero plaque assays were performed using supernatant collected at 24 hpi to quantify viral titers. Relative to vehicle (DMSO)-treated, CHIKV-infected controls, ABT-888 (*P* < 0.001, 10 μM and 1 μM), Olaparib (*P* < 0.5, 0.1 μM; *P* < 0.01, 0.01 μM), 413A (*P* < 0.01, 1 μM), and 413B (*P* < 0.05, 0.1 μM) significantly increased CHIKV titers in C8-D1A astrocytes, thus indicating PARP1 and PARP2-mediated negative regulation of CHIKV replication in astrocytes (Fig. 5A). No changes in PJ-34-treated C8-D1A viral titers were observed. In contrast, ABT-888 (*P* < 0.0001, 10 μM and 1 μM; *P* < 0.01, 0.1 μM; *P* < 0.05, 0.01 μM), Olaparib (*P* < 0.0001, 10 μM; *P* < 0.05, 0.1 μM; *P* < 0.01, 0.01 μM), PJ-34 (*P* < 0.01, 10 μM; *P* < 0.05, 0.1 μM), 413A (*P* < 0.001, 10 μM), and 413B (*P* < 0.0001, 10 μM and 1 μM) significantly decreased viral titers in NSC-34 neurons (Fig. 5B). Therefore, all PARPs examined appear to negatively regulate CHIKV replication in C8-D1A astrocytes, with inhibition of PARP1 and PARP2 resulting in the highest viral titers (Fig. 5). In contrast, inhibition of PARP1 and PARP2 decreased CHIKV titers in neurons, indicating positive regulation of virus replication. Importantly, such changes in viral replication dynamics cannot be attributed to changes in host viability (Fig. S3A and B).

### PARP1-mediated ADP-ribosylation during infection is critical for viral replication in neurons

Based on our previous findings (24) and above data, both MARylating and PARylating ARTs are crucial in promoting or impeding CHIKV replication in neurons or astrocytes, respectively. In neurons, drug inhibitors targeting PARP1 and PARP2 (ABT-888, Olaparib) most dramatically modified CHIKV titers at the lowest drug concentrations tested (Fig. 5B). Therefore, we further investigated PARP1, or the main ART family member postulated to be responsible for the majority of PARylation. *In vitro* CHIKV infection is associated with up to a 95% increase in DNA damage, which is associated with caspase 3-mediated PARP1 cleavage and activation, followed by subsequent PARP1 PARylation of substrates involved in DNA repair (65, 66). During DNA damage, about 85%–90% of cellular PARP activity is attributed to PARP1, with 10%–15% contributed by PARP2, while the remaining activity is shared by other PARP enzymes (66). Based on these observations, in addition to the observation of diminished CHIKV titers in PARP1 inhibitor-treated NSC-34 cells, we postulated that the increased ADP-ribosylation in CHIKV-infected neuronal cells, while limited in infected astrocytes (Fig. 4A), may be mediated by PARP1.

**Fig 6:**
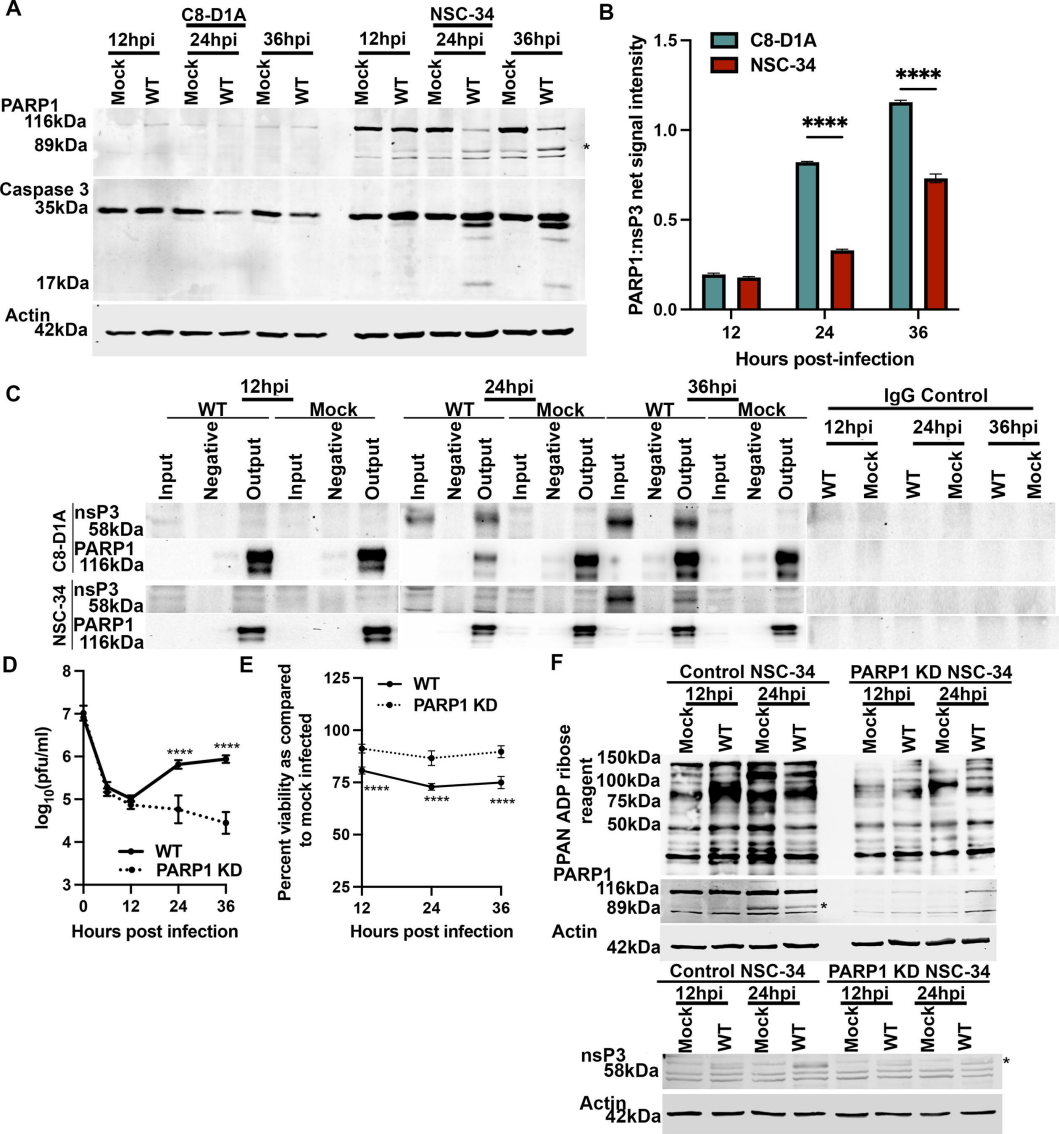
PARP1 activation, CHIKV nsP3 interaction, and ADP-ribosylation in astrocytes and neurons. (**A**) C8-D1A and NSC-34 cells were either mock-infected or infected with CHIKV WT (MOI 5); collected lysates were subjected to Western blotting and probed against PARP1, caspase 3 and β-actin. A representative image from three biological experiments is shown. (**C**) CHIKV nsP3 protein was pulled down by antibody-mediated co-immunoprecipitation using antibodies against nsP3 (“output”) or rabbit IgG control (“negative”). Pulled-down products were then analyzed by Western blotting to probe for both nsP3 and PARP1 in C8-D1A and NSC-34 neurons. A representative image from three biological experiments is displayed. (**B**) Densitometric analysis was additionally performed to compare PARP1 to nsP3 net signal intensity. ****P < 0.0001 (C8-D1A vs NSC-34). (**D**) NSC-34 PARP1 KD and corresponding WT cells were either mock-infected or infected with CHIKV WT (MOI 5), supernatants were collected (*n* = 3 biological samples per time point), and viral titration was quantified through Vero plaque assays. Data represent the average ± SD from three independent experiments. ****P < 0.0001 (NSC-34 PARP1 KD versus WT). (**E**) NSC-34 PARP1 KD and corresponding WT cells were either mock-infected or infected with CHIKV WT (MOI 5); at corresponding hpi, CellTiter Glo reagent was added and cell viability was measured per manufacturer’s instruction (*n* = 8 biological samples per time point). Data represent the average ± SD. ****P < 0.0001 (WT vs NSC PARP1 KD). (**F**) Lysates from NSC-34 PARP1 KD and corresponding WT cells mock-infected or infected with CHIKV WT (MOI 5) were subjected to Western blotting and probed against pan-ADP-ribose reagent, PARP1, nsP3, and β-actin. A representative image from three biological experiments is presented.

To investigate this hypothesis, we analyzed PARP1 expression in both NSC-34 neurons and C8-D1A astrocytes upon infection. Cell lysates collected at different hpi were subjected to Western blot analysis for PARP1 and caspase-3 expression (Fig. 6A), the latter expected to cleave native PARP1 (116 kDa) into an 89-kDa active form and 24-kDa inactive form. Native 116-kDa PARP1 expression was dramatically lower in C8-D1A cells than NSC-34 cells; furthermore, no infection-induced increase was observed in astrocytes. In contrast, PARP1 activation, as evident by visible bands at 89 kDa, occurred in WT-infected NSC-34 cells at 24 hpi and 36 hpi. Notably, PARP1 cleavage was also observed in mock-infected cells at 36 hpi, likely due to cellular stress from overgrowth and media starvation. Consistently, uncleaved (inactive) 35-kDa pro-caspase-3 expression is observed in both infected and mock-infected NSC-34 neurons and C8-D1A astrocytes (Fig. 6A). However, presence of the 17-kDa subunit, a result of caspase-3 cleavage, in infected NSC-34 cells is indicative of caspase-3 activation. Such caspase-3 activation temporally corresponds with PARP1 cleavage and appears to be specific to CHIKV-infected neurons, but not astrocytes. Therefore, we postulated that cell type-dependent changes in both PARP1 activation and nsP3-mediated type I IFN expression observed in neurons versus astrocytes may be attributed directly to PARP1–nsP3 interaction frequency. We have previously demonstrated PARP1–nsP3 C-terminal domain, but not N-terminal MD, interaction in SINV infection of NSC-34 cells (67). Therefore, we infected NSC-34 and C8-D1A cells with mock reagent or CHIKV WT, harvested protein lysates at designated hpi, and proceeded to pull down nsP3 protein via co-immunoprecipitation experiments. Results indicated that PARP1 co-immunoprecipitates with nsP3 in both CHIKV-infected C8-D1A astrocytes and NSC-34 neurons, but not mock-infected cells, as expected (Fig. 6; Fig. S4). Densitometric analyses demonstrated a significantly higher PARP1–nsP3 signal ratio in astrocytes than neurons (Fig. 6B), indicating enhanced PARP1 interaction with CHIKV nsP3 in astrocytes.

Given the observed role of PARP1 in neuronal cells, we employed a genetic depletion strategy using shRNA to reduce constituent PARP1 levels in NSC-34 cells. Compared with WT counterpart cells, CHIKV titers in PARP1 knock down (KD) NSC-34 cells were drastically reduced at 24 and 36 hpi (*P* < 0.0001; Fig. 6D) with minimal cytopathic effect (*P* < 0.0001; Fig. 6E). Western blot analysis of cell lysates confirmed the depletion of PARP1 in mock-infected cells, with PARP1 only detected in CHIKV-infected KD cells at 24 hpi (Fig. 6F). However, no PARP1 cleavage was observed in PARP1 KD cells, regardless of infection status, as evident by lack of 89-kDa bands. ADP-ribosylation, which increased in WT NSC-34 cells upon CHIKV infection at 12 hpi relative to mock-infected cells (Fig. 4A), appeared much greater in signal intensity relative to PARP1 KD NSC-34 cells, thus indicating PARP1 involvement with ubiquitous cellular ADP-ribosylation activity, as expected. Considering the aforementioned constituent PARP1 depletion in NSC-34 decreasing CHIKV titers, this indicates that PARP1 activation, and thus its PARylation activities, may be critically important during CHIKV replication in neuronal cells.

## DISCUSSION

**Fig 7:**
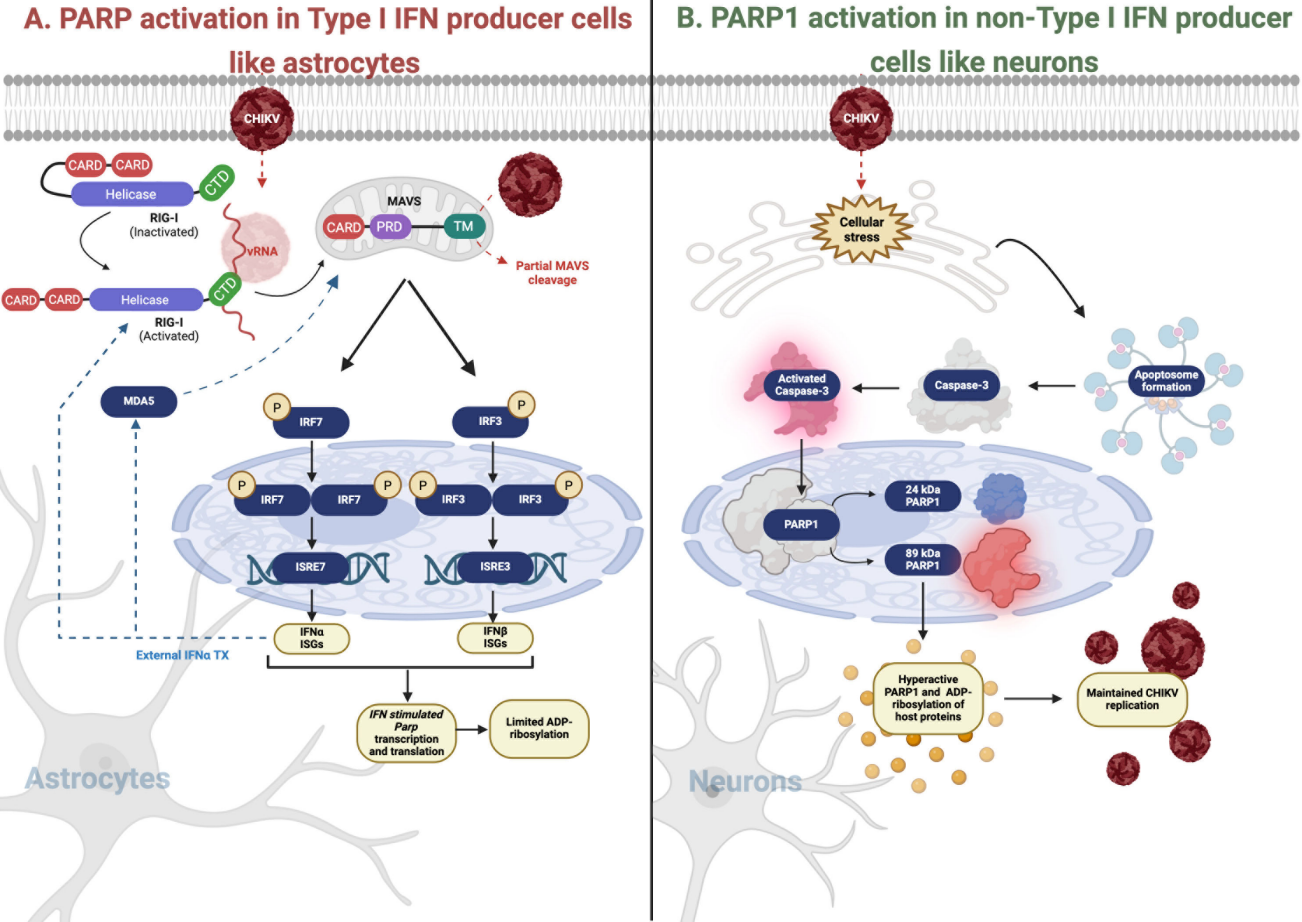
CHIKV activates type I interferon expression and PARP1 activation in a cell type-dependent fashion in neurons versus astrocytes. (**A**) CHIKV infection in astrocytes activates RIG-I expression, MAVS activation, IRF3 phosphorylation, and subsequent IRF3 nuclear translocation, followed by type I IFN expression and ISG gene expression. While *Parp1* transcription is activated, no upregulation in PARP1 expression is observed. Limited global ADP-ribosylation, a known host antiviral response, is thus observed, and CHIKV can therefore replicate to much higher titers, eventually resulting in astrocytic host death. The CHIKV nsP3 MD regulates part of this pathway by partially inhibiting type I IFN expression through an unknown mechanism. (**B**) CHIKV infection in neurons does not activate RIG-I expression and therefore no type I IFN production, but rather activates caspase-3-mediated PARP1 cleavage and subsequent activation, followed by PARP1-mediated hyper ADP-ribosylation of host proteins. As a result, CHIKV replication is maintained in a persistent fashion in neurons. (Created using https://BioRender.com).

In our current study, we strived to elucidate cell type-dependent innate immune responses to CHIKV infection elicited by neurons versus astrocytes, the latter known to be the main CHIKV CNS target (38, 52). Using both human and murine astrocytic and neuronal *in vitro* models, we characterized the innate immune responses elicited by astrocytes and neurons against CHIKV infection and demonstrated that CHIKV infection activates RIG-I viral RNA sensors and, subsequently, type I IFN expression in astrocytes; interestingly, neither RIG-I activation nor type I IFN production was observed in neurons. In astrocytes, type I IFN dynamics were further found to be regulated by the nsP3^MD^, as Y114A (increased ADPr binding propensity, but lower ADPr hydrolase capacity) and G32S (lower ADPr binding and hydrolase capacity) nsP3^MD^ mutations were associated with upregulation of IFNα and IFNβ expression in human astrocyte cells *in vitro*. Further exploration into the mechanism behind RIG-I activation and subsequent type I IFN expression in CHIKV-infected astrocytes revealed increased IRF3 phosphorylation, ensuing nuclear translocation, and thus subsequent IFNα and IFNβ induction in CHIKV-infected astrocytes (Fig. 7A). In neurons, neither phosphorylation nor subsequent nuclear translocation of IRF3 was observed; instead, neuronal CHIKV infection uniquely resulted in caspase-3 activation, PARP1 activation, and thus PARP1-mediated changes in global ADP-ribosylation (Fig. 7B).

Interestingly, inhibition of PARP1 and PARP2 significantly decreased viral replication in neurons, whilst constitutive *Parp1* shRNA knockdown in NSC-34 cells significantly hampered CHIKV titers, indicating PARP1 serving as a positive regulator of CHIKV replication in neurons. In contrast, neither caspase-3 nor PARP1 activation was observed in infected astrocytes, with no subsequent changes in global ADP-ribosylation being present, regardless of infection status. Furthermore, PARP1 inhibition increased viral titers in astrocytes. CHIKV infection has previously been shown to induce up to 95% total DNA damage in non-neuronal and non-astrocytic cell lines and is known to activate DNA damage response pathway checkpoint kinases Chk1 and Chk2 (65). Such DNA damage is typically associated with caspase 3-mediated PARP1 activation, cytosolic NAD+ depletion, subsequent PAR accumulation within the nucleus and, consequently, either cellular quiescence, viral persistence, or cell death (68). Since PARP1 activation is known to induce apoptosis-inducing factor-dependent programmed cell death (69) and that PARP1 is cleaved in CHIKV-infected neurons, it remains possible that PARP1 may induce cell death or, in the case of mature, differentiated neurons, be involved with noncytolytic clearance and viral persistence (70–73). However, PARP1 has yet to be studied in the context of CHIKV infection, let alone between infected cerebral cell types. Previous *ex vivo* studies have demonstrated that PARP1 activation is both brain region- and cell type-specific, with PAR induction occurring within specific astrocytic and neuronal subtypes in the stratum radiatum and hippocampus, but minimally within the prefrontal cortex (74). Therefore, in order to improve understanding of the role of PARP1 in host cell death during CHIKV infection, future experiments involving various CHIKV-infected neuronal subtypes are needed in order to examine cell type-dependent, CHIKV-mediated PARP1 activation, NAD^+^ cytosolic depletion, PAR nuclear accumulation, as well as CHIKV replication, viral RNA persistence, and host viability.

Our previous research found enhanced overall viral replication and translation of genomic viral proteins such as nsP3 in astrocytes than neurons (36). We have additionally shown that PARP1 coimmunoprecipitates with SINV nsP3 and is present within viral replication complexes in infected NSC-34 cells (75). Since PARP1 is activated only in CHIKV-infected neurons, but not astrocytes, and positively regulates CHIKV replication in neurons, PARP1 may represent a cell type-dependent host factor. Further identification of host substrates PARylated by active PARP1 in neurons, as indicated by increased global ADP-ribosylation in our current study, is thus necessary to understand the role of PARP1 in viral replication regulation, host DNA repair, and thus potentially CHIKV persistence. Though we observed improved CHIKV replication in ABT-888-treated astrocytes, as well as lack of PARP1 activation in infected astrocytes, it remains unknown how PARP1 is involved during CHIKV infection in astrocytes. One possible mechanism may involve nsP3, which is generated in higher quantities in astrocytes than neurons (38) and may directly cleave ADPr from auto-modified PARP1, thus diminishing PARP1 DNA binding affinity, repair machinery recruitment, and subsequent host survival. Further investigation must be carried out to determine the role of PARP1 in astrocytes and to characterize PARP1 automodification, as well as nsP3^MD^-mediated post-translational modification of PARP1 or substrates recruited by PARP1 in a MARylation-dependent manner such as sirtuin 6 (SIRT6), a negative regulator of RLR pathway activation in *in vitro* dengue virus models (76, 77). Auto-modification of PARP1 enables PARP1-mediated recruitment of DNA repair factors, of which encourages proliferation by eschewing G1/G2 cell cycle arrest (78, 79), and may thus prompt viral persistence. PARP1 activation occurring in neurons, but not astrocytes, is indicative of non-cytolytic clearance and thus such CHIKV persistence. Further examination into how PARP1 impacts CHIKV-mediated host DNA damage and subsequent neuronal viability during CHIKV infection is thus needed. PARP1 has also been found to facilitate type I IFN receptor (IFNAR) degradation (80), termination of cGAS-genomic DNA binding (81), and improvement of influenza virus replication (80, 82). However, considering that PARP1 knockdown reduces CHIKV fitness in neurons, yet improves viral replication in astrocytes, it remains possible that PARP1 activation mediates neuronal survival by mitigating IFN production via inhibition of IFNAR degradation, as IFN production is known to induce neuronal death (83). PARP1 may additionally instigate genomic DNA repair, thus enabling improved neuronal cell survival, which is associated with alphavirus persistence (84). PARP1 inhibition of the cGAS pathway remains an unlikely route of PARP1-mediated survival, as no changes in *Cgas* or *Sting* expression in mock-infected or WT-infected NSC-34 neurons were observed.

Although most neurovirulent viruses, including alphaviruses, primarily target neurons, CHIKV displays tropism towards astrocytes (38). However, the underlying mechanisms behind such astrocyte tropism and subsequent neurovirulence involving faster and higher viral replication in astrocytes versus other CNS cells remain unclear. Astrocytes are the most abundant stromal cells within the brain and, as is the case in most neurotropic viral infections, are the main source of type I IFN predominantly through IFNβ-mediated activation of innate immune pathways (85–89). Type I IFNs in the CNS during infection can also be from the infiltration of activated innate immune cells (90). Innate immune responses mediated by type I IFNs are crucial for controlling infections in the cerebral environment. Most cells in the CNS are efficient responders to type I IFN signaling (91). For neurons, however, the ability to respond to or produce type I IFN during infections, as well as the resulting outcome, may be directly linked to their maturity. For example, immature neurons have a reduced competence to activate antiviral defenses (92). NSC-34 cells, a neuroblastoma-spinal cord hybrid that displays a multipolar neuron-like phenotype resembling a developing motor neuron (54), shows no activation of PRRs or subsequent activation of the IRF3/7 transcription factors during infection. As a result, NSC-34 cells do not produce type I IFNs (24). Therefore, CHIKV infection of astrocytes may occur to shut down early type I IFN production, of which is associated with reduced CHIKV replication and persistence (93). Active PARP1 imposes a substantial burden on cellular NAD^+^ levels, a critical metabolic factor (94, 95). While exact cytosolic NAD^+^ or nuclear PAR levels within each cell lines studied remains unknown, the cell type-dependent expression activation of PARP1, along with the type I IFN response and the ADP-ribosylated protein states in both cell types, likely influence various stages of viral replication. Moreover, viruses including CHIKV counteract the NAD-mediated host defense mechanism through MD-mediated replication. Interestingly, despite the significant modulation of IFN-stimulated PARPs during infection, only a minor change in the total ADP-ribosylation of astrocytic proteins was observed (Fig. 4A). This may be attributed to increased PARP1–nsP3 interaction frequency compared with neurons (Fig. 6B and C). In neurons, IFN-stimulated PARPs were not modulated, though an intense increase in total ADP-ribosylated proteins was observed. This suggests that IFN-stimulated PARPs, which are predominantly MARylating enzymes, may not contribute substantially to the overall aggregated ADP-ribosylation of neuronal proteins, but could be regulated at a more subtle, sub-molecular level. Furthermore, the transcriptional activation of IFN-stimulated PARPs in both astrocytes and neurons appears to be tightly regulated.

One of the major limitations of this study was that the experiments were conducted in cell lines and may not reflect cell type-dependent CHIKV interactions within the CNS; therefore, investigating these findings in primary neural cells is warranted. In summary, we demonstrated cell type-dependent innate immune responses elicited by neurons and astrocytes upon CHIKV infection *in vitro* (Fig. 7). Upon CHIKV infection, astrocytes elicited a RIG-I-mediated type I IFN response involving IRF3 phosphorylation and nuclear translocation, a pathway partially regulated by the nsP3^MD^ through an unknown mechanism. In neurons, neither RIG-I activation nor type I IFN expression occurred following CHIKV infection. Instead, CHIKV infection activated caspase 3, PARP1, and subsequently induced global ADP-ribosylation. Elucidation of both pathways is an essential step in understanding how CHIKV elicits cell type-specific immune responses, and thus how these two cell types contribute to the damaging inflammatory responses observed in CHIKV meningitis, as well as CHIKV persistence.

## Data Availability

All data generated or analyzed during this study are included in this article. Further inquiries can be directed to the corresponding author.

## SUPPLEMENTAL MATERIAL

Supplemental material spectrum.04178-25-s0001.pdf Supplemental methodology, Table S1, and Fig. S1 to S4.

ASM does not own the copyrights to Supplemental Material that may be linked to, or accessed through, an article. The authors have granted ASM a non-exclusive, world-wide license to publish the Supplemental Material files. Please contact the corresponding author directly for reuse.
