# Supplementary material for: Cell type-dependent induction of type I interferon and PARP1 activation in astrocytes and neurons during chikungunya virus infection
Source: Microbiol Spectr. 2026 Jun 24;14(8):e04178-25. doi: 10.1128/spectrum.04178-25 (PMC13435702; doi:10.1128/spectrum.04178-25)
Supplement: Supplementary file 1 [file spectrum.04178-25-s0001.pdf]

Supplementary files

Chemistry

General: 1H NMR spectra were recorded on a Bruker DPX spectrometer at 400 MHz. Chemical shifts are reported as parts per million (ppm) downfield from an internal tetramethylsilane standard or solvent references. For air- and water-sensitive reactions, glassware was flame- or oven-dried prior to use and reactions were performed under argon. Dimethylformamide was dried using the solvent purification system manufactured by Glass Contour, Inc. (Laguna Beach, CA). All other solvents were of ACS chemical grade (Fisher Scientific) and used without further purification unless otherwise indicated. Commercially available starting reagents were used without further purification. Analytical thin-layer chromatography was performed with silica gel 60 F254 glass plates (SiliCycle). Flash column chromatography was conducted self-packed columns containing 200-400 mesh silica gel (SiliCycle) on a Combiflash Companion purification system (Teledyne ISCO). High performance liquid chromatography (HPLC) was performed on a Varian Prostar 210 (Agilent) using Polaris 5 C18-A columns (Analytical: 150 x 4.6 mm, 3 μm; Preparative: 150 x 21.2 mm, 5 μm) (Agilent). All final products were ≥95% pure as assessed by analytical HPLC (mobile phase A: 0.1% formic acid (aq), mobile phase B: 0.1% formic acid in acetonitrile; flow rate = 1.0 mL/min; conditions: pre-run A = 90 % B = 10%, 10 min A = 5% B = 95%, 11 min A = 5% B = 95%, 13 min A = 90% B = 10%; UV-Vis detection: λ1 = 254 nm, λ2 = 220 nm. Retention times (Rt) refer to UV = 254 nm.

2-(4-(2-Fluoro-5-((4-oxo-3,4-dihydrophthalazin-1-yl)methyl)benzoyl)piperazin-1-yl)benzonitrile, **413A**: 2-fluoro-5-((4-oxo-3,4-dihydrophthalazin-1-yl)methyl)benzoic acid (Ambeed) (50 mg, 0.17 mmol) (, TBTU (60 mg, 0.19 mmol) and 2-(piperazin-1-yl)benzonitrile (Oakwood Chemical) were added to a flame dried flask and dissolved in anhydrous DMF/DCM (1:10) (5 mL). DIPEA (0.06 mL, 0.34 mmol) was added under argon and the reaction stirred at RT for 18 h. The product was then precipitated in water (100 mL) and collected by vacuum filtration. from 2-(piperazin-1-yl)benzonitrile (35 mg, 0.19 mmol). Yield: 60 mg (75%). 1H NMR (400 MHz, DMSO-d6) δ 12.61 (s, 1H), 8.30 – 8.23 (m, 1H), 7.98 (d, J = 8.0 Hz, 1H), 7.90 (td, J = 7.6, 1.5 Hz, 1H), 7.83 (t, J = 7.5 Hz, 1H), 7.74 (dd, J = 7.7, 1.7 Hz, 1H), 7.67 – 7.58 (m, 1H), 7.42 (ddd, J = 16.3, 6.1, 2.7 Hz, 2H), 7.25 (t, J = 9.0 Hz, 1H), 7.20 – 7.12 (m, 2H), 4.35 (s, 2H), 3.80 (d, J = 5.5 Hz, 2H), 3.19 (d, J = 12.6 Hz, 3H), 3.05 (d, J = 6.4 Hz, 2H), 2.66 (s, 1H). MS m/z [M-H]- for $C_{27}H_{22}FN_5O_2$: 465.7. Rt = 9.27 min.

1H NMR for 413A with chemical structure:

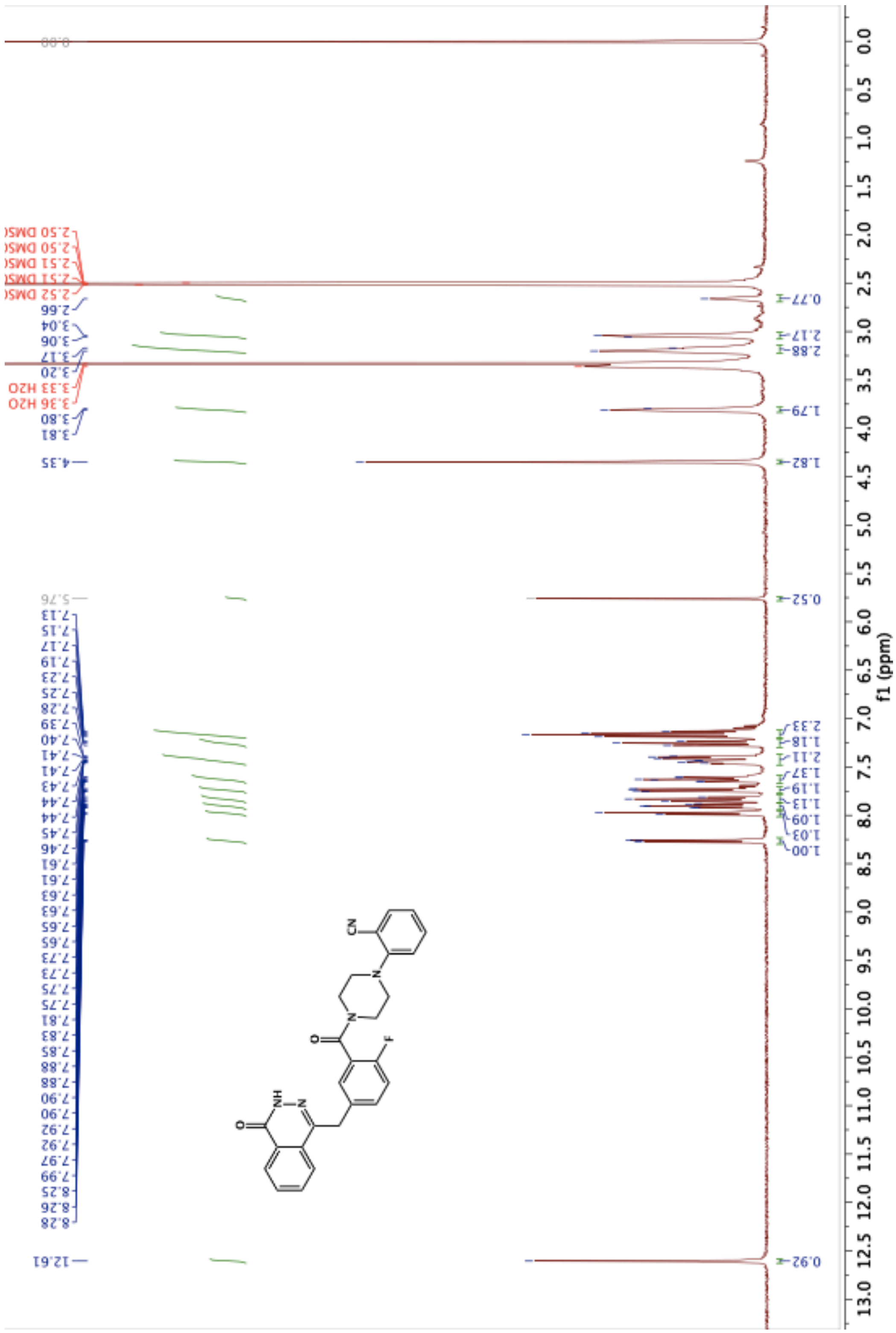

Table S1. Selectivity data for PARP inhibitors 413A and 413B

	PARP1	PARP2	PARP3	$PARP4_{brct-cat}$	$PARP5b_{cat}$	PARP6	PARP7
**413A**	0.015±0.001	0.015±0.01	0.12±0.0002	0.060±0.002	5.0±0.09	0.053±0.02	>10
**413B**	0.021±0.004	0.028±0.005	0.32±0.002	0.35±0.002	2.52±0.2	0.18±0.05	0.014±0.002
	**PARP8**	**$PARP10_{cat}$**	**PARP11**	**$PARP12_{cat}$**	**$PARP14_{cat-wwe}$**	**$PARP15_{cat}$**	**PARP16**
**413A**	0.79±0.02	>3	>3	>3	>10	1.8±0.6	>3
**413B**	4.7±1	0.86±0.3	0.46±0.02	>3	>10	2.0±0.3	4.3±1

Selectivity data for 413A and 413B against individual PARPs or specific PARP domains. PARP inhibitor family wide screening (PASTA) was performed as previously mentioned [58,59]. Inhibitor dose-response curves were fitted using linear regression in GraphPad Software. The mean IC_{50} for each compound was calculated from atleast three independent assays and presented in μM. IC_{50} data for 413B were adapted from [57]. Abbreviations: BRCT–CAT, BRCA1 C-terminus–catalytic domain; CAT, catalytic domain; CAT–WWE, catalytic–WWE domain.

Supplementary Figures

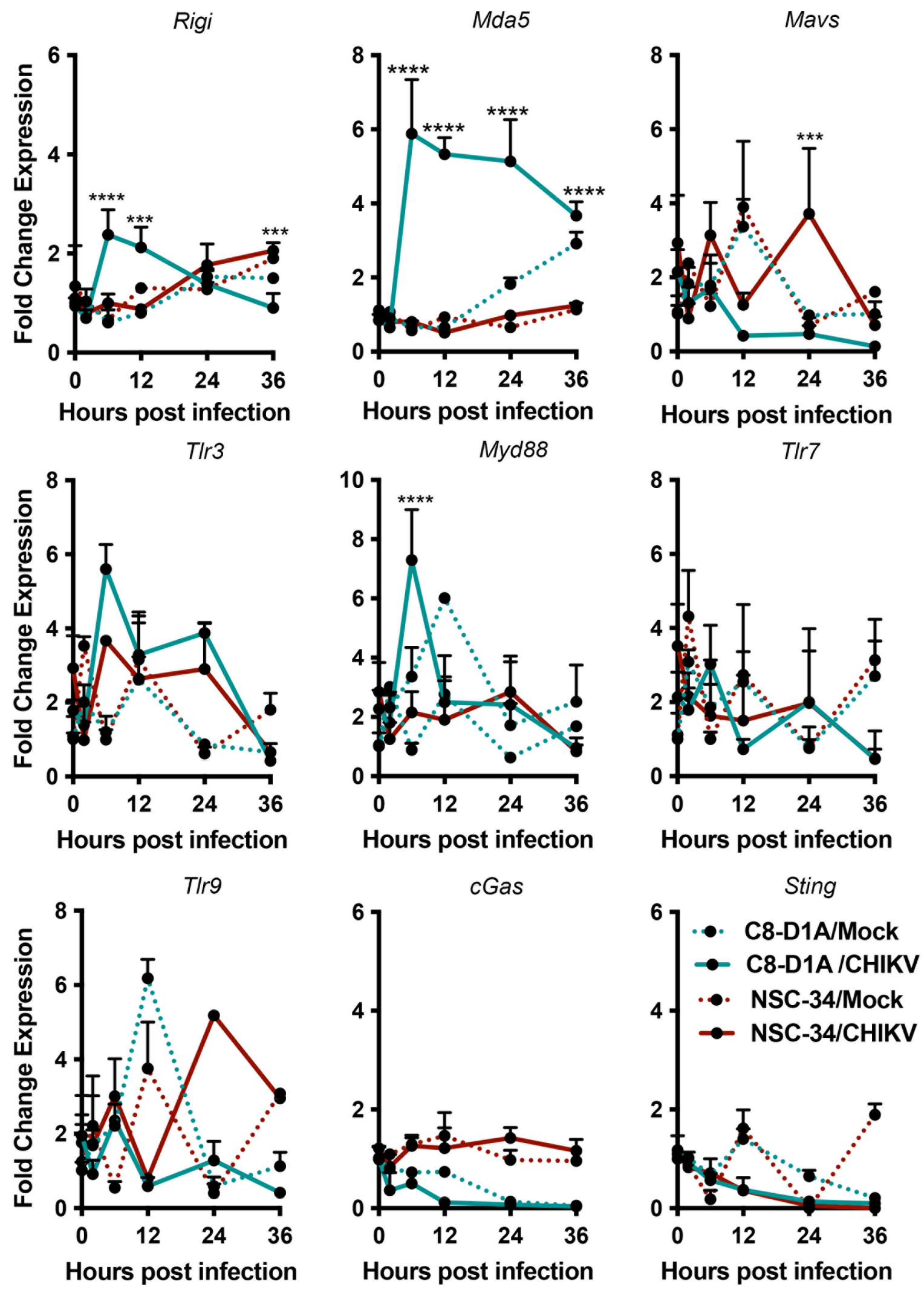

Fig. S1. Expression of RLR, *TLR* and other innate immune mRNAs in CHIKV-Infected Neurons and Astrocytes. (A) NSC-34 and C8-D1A cells were either mock-infected or infected with WT CHIKV at an MOI of 5 and RNA was isolated, and cDNA synthesized (n=3 biological samples per time-point). Gene expression for Rigi, Mda5, Mavs, Myd88, *Tlr3, Tlr7, Tlr9*, *cGas* and *Sting* was determined by real-time PCR and C_t values normalized to *Gapdh/RPS29.* Fold change in expression was calculated by comparison to the 0-hour mock-infected samples. Data show the average ± SD. ***P < 0.001, ****P < 0.0001 (C8-D1A inf vs NSC-34 inf).

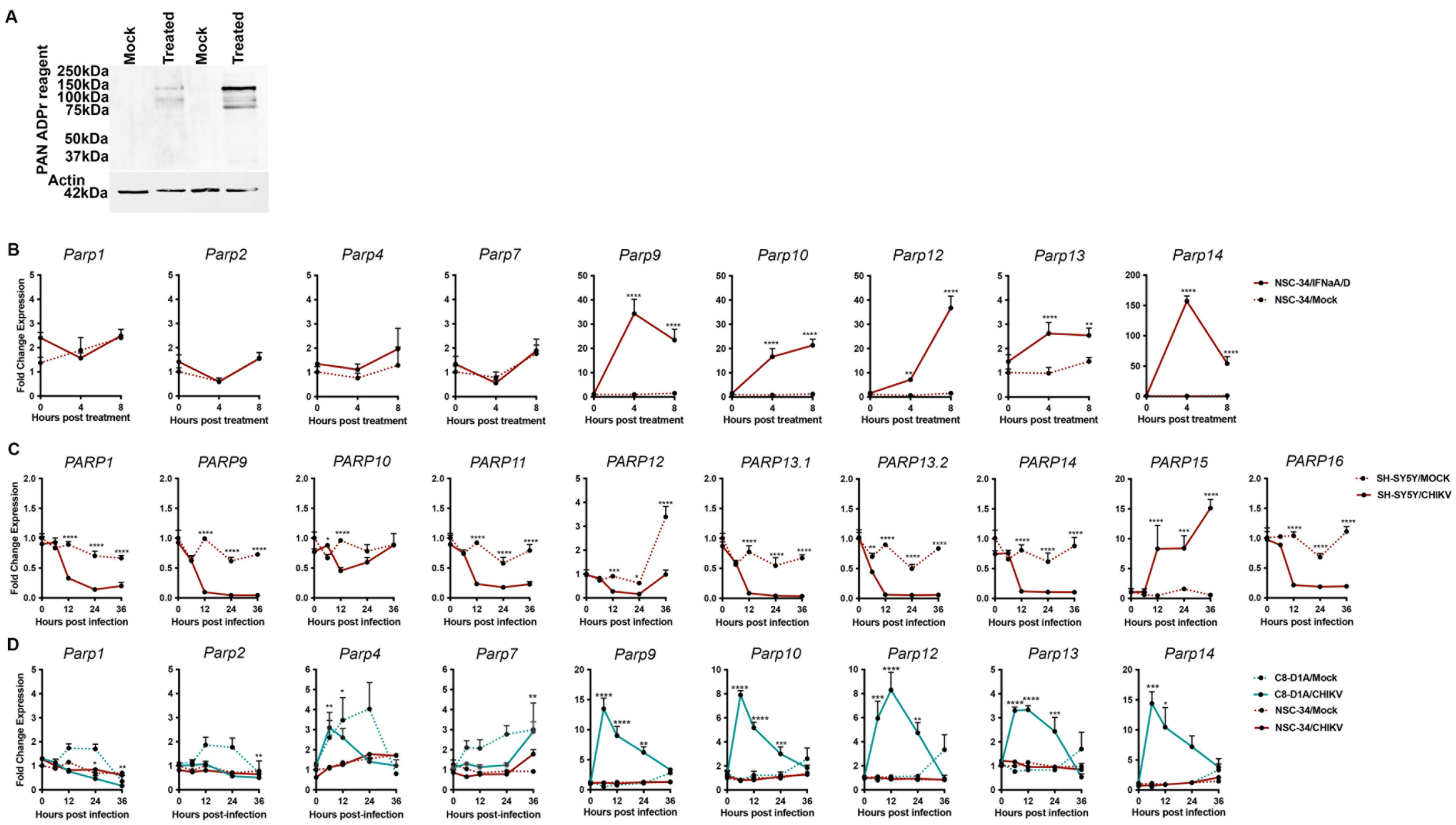

Fig. S2. ADP-ribosylation and PARP modulation in Astrocytes and Neurons. (A) NSC-34 and C8-D1A cells were either mock-treated or treated with 0.3mM of H_2O_2 and the lysates were analyzed through western blotting. (B) RNA was isolated from mock treated and 500U of IFNα A/D treated cells and qRT-PCR was performed for *Parp*1, 2, 4, 7, 9, 10, 11, 12 and 14 to determine gene expression. C_t value for each transcript was normalized to *Gapdh.* Fold change in expression was calculated relative to 0-hour mock-treated samples. Data represents the average ± SD. (C) RNA was isolated from mock infected and CHIKV (WT) infected SH-SY5Y cells and qRT-PCR was performed for *PARP*1, 9, 10, 11,12, 13.1, 13.2, 14, 15 and 16 to determine the gene expression. C_t value for each transcript was normalized to *GAPDH.* Fold change in expression was calculated relative to 0-hour mock-infected samples. Data represent the average ± SD. ****P < 0.0001, *** P < 0.001(Mock vs CHIKV). (D) RNA was isolated from mock infected and

CHIKV (WT) infected C8-D1A cells and qRT-PCR was performed for *Parp*1, 2, 4, 7, 9, 10, 11, 12 and 14 to determine gene expression. C_t value for each transcript was normalized to *Gapdh.* Fold change in expression was calculated relative to 0-hour mock-infected samples. Data represent the average ± SD. ** *P* < 0.01, **P* < 0.05 (C8-D1A CHIKV vs NSC-34 CHIKV).

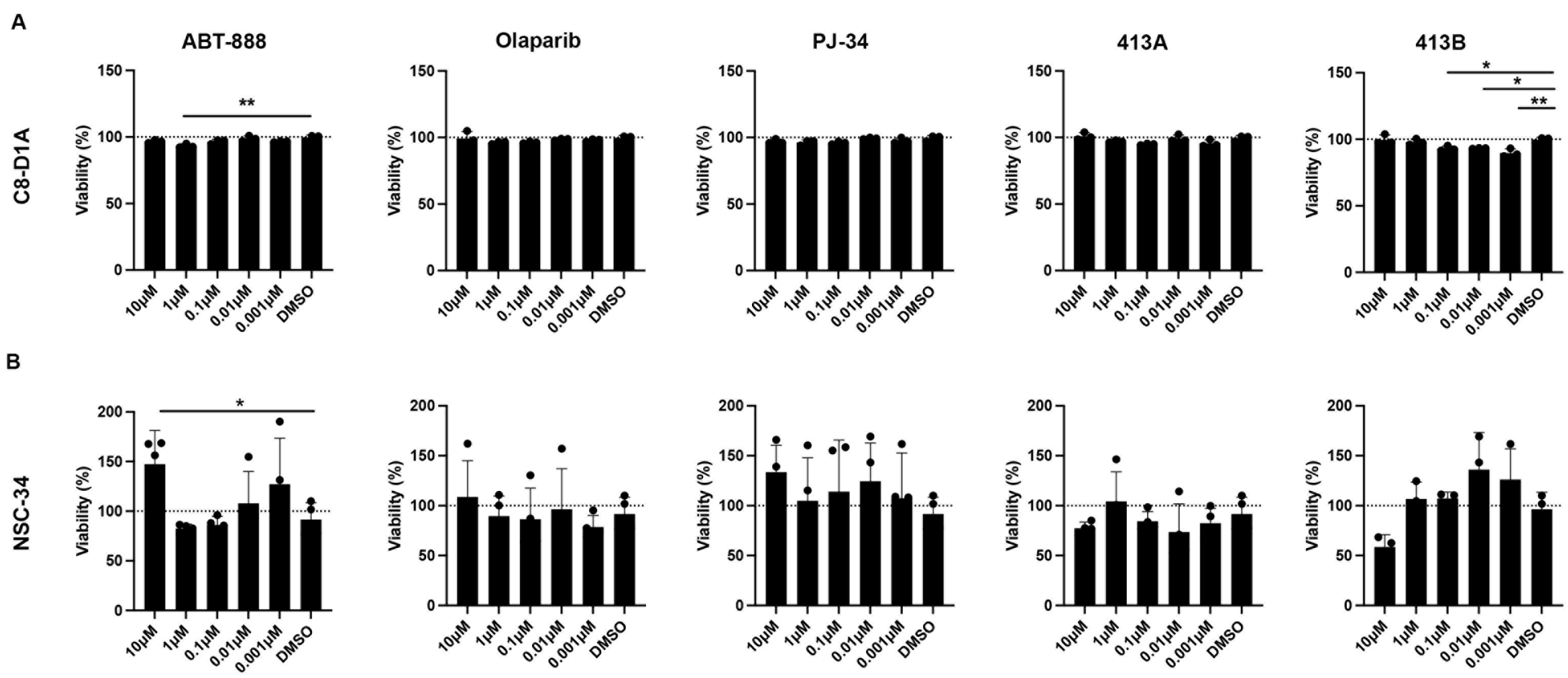

Fig. S3. Cytotoxicity of PARP inhibitors in Astrocytes and Neurons upon treatment. (A) C8-D1A and (B) NSC-34 cells were treated with 0.1–10 μM of PARP1 and PARP2 specific inhibitors ABT-888, Olaparib and PJ-34 and broad PARP specific inhibitors 413A and 413B for 24 hpi (n=3 biological samples per time-point). Data represent the average ± SD. *P < 0.05, **P < 0.01, ***P < 0.001

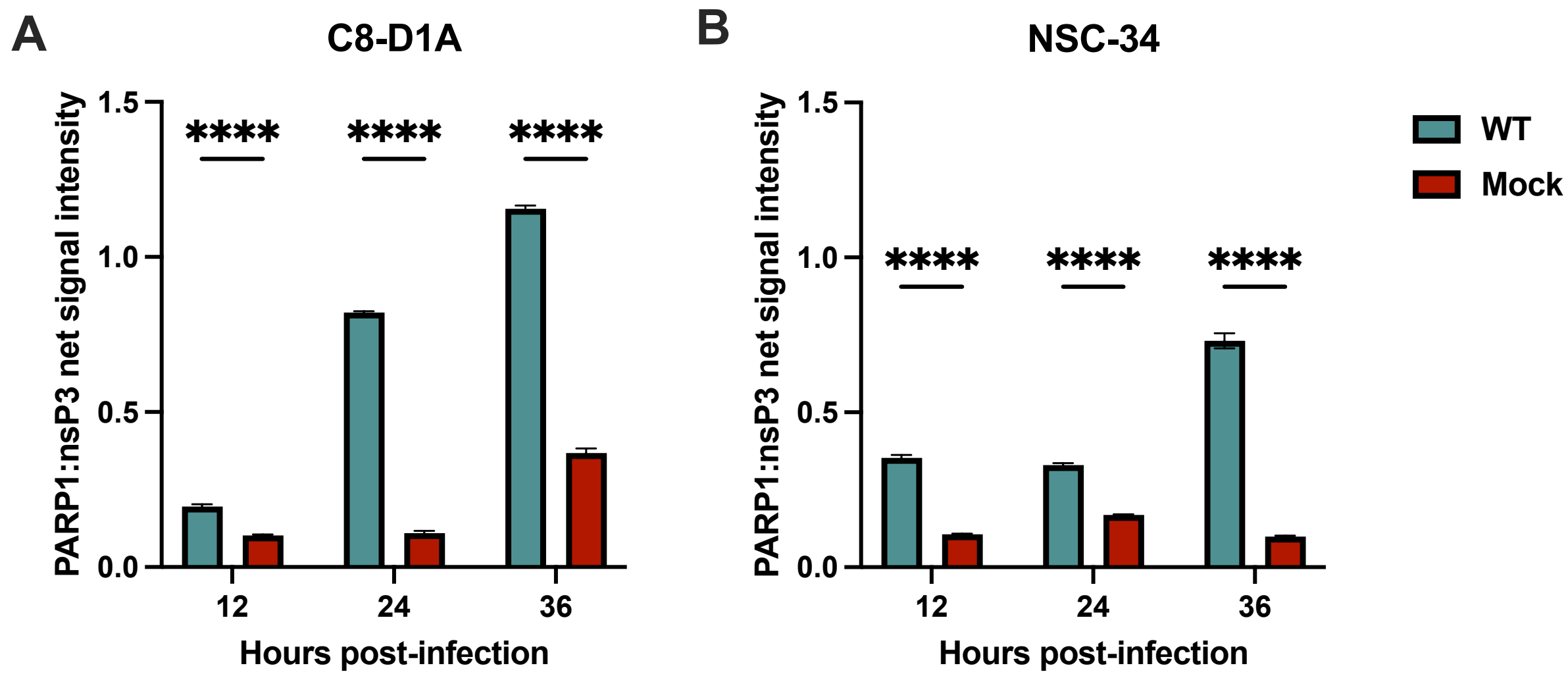

Fig. S4. Interaction of PARP1 and nsP3. Densitometric analysis of band intensities from the pull-down assay. A representative image from n=3 blots is shown in Figure 6C. Densitometric analysis was performed in CHIKV-infected versus mock-infected (A) C8-D1A and (B) NSC-34 cells to compare the net signal intensities of both PARP1 and nsP3 bands at the indicated hours post-infection.
